# A novel method for approximate solution of two point non local fractional order coupled boundary value problems

**DOI:** 10.1371/journal.pone.0326101

**Published:** 2025-07-02

**Authors:** Lahoucine Tadoummant, Hammad Khalil, Rachid Echarggaoui, Sarah Aljohani, Nabil Mlaiki

**Affiliations:** 1 Department of Mathematics, Ibn Tofail University, Kenitra, Morocco; 2 Department of Mathematics, University of Education, Lahore (Attock Campus), Pakistan; 3 Department of Mathematics, Ibn Tofail University, Kenitra, Morocco; 4 Department of Mathematics and Sciences, Prince Sultan University, Riyadh, Saudi Arabia; University of Porto Faculty of Engineering: Universidade do Porto Faculdade de Engenharia, PORTUGAL

## Abstract

The aim of this paper is to investigate the solution of fractional-order partial differential equations and their coupled systems. A novel method is proposed, which effectively handles these problems under two-point non-local boundary conditions. The method is based on shifted Legendre polynomials, and some new operational matrices for these polynomials are constructed. In order to convert the partial differential equation together with its nonlocal boundary condition these matrices play important role. The matrices are used to convert the fractional-order derivatives and integrals, as well as the non-local boundary conditions to a system of algebraic equations. The convergence of the proposed method is rigorously analyzed and supported by a range of computational examples. The results obtained with the proposed method shows that the absolute and relative errors decreased for both the solutions *X* and *Y* as the parameter *M* increases. A significant reduction in both error types is observed, with the relative error |*X*_*r*_| decreasing from approximately 10^−1^ to 10^−8^. We observed that the convergence rates lie in the range of 1.016 to 1.497 for |*X*_*r*_|, and 0.985 to 1.451 for |*Y*_*r*_|. These results confirm the high precision and exponential convergence behavior of the proposed numerical method. All simulations are performed using MATLAB to validate the proposed approach. The algorithm is presented as pseudo code in the article. The MATLAB codes used for the simulation of the algorithm is presented as supplementary material.

## Introduction

Fractional-order derivatives and integrals have opened a new avenue for modeling various physical phenomena. These models often provide more accurate representations than their integer-order counterparts. Recent advances highlight their role in modeling biochemical dynamics. In [1], Akgul *et al*. offer a detailed analysis of fractional-order nonlinear systems in biochemical reactions. Elwakil *et al*. emphasize emerging trends in this domain [2]. Some notable contributions can also be found in [3–6]. Fractional calculus have also gained attention in heat dynamics. Dzielinski *et al*. discuss modeling heat flow in a non-uniform rigid beam, showing the efficiency of fractional models through experiments [7]. A model for heat conduction in a rectangular plate appears in [12], and phase lag effects are explored by Dahab *et al*. in [13]. Heat transfer in heterogeneous media is further studied in [14]. Chen *et al*. model building heat dynamics in [8], while Dlugosz *et al*. apply similar models to building structures [9]. The reader can find a detialed application of fractional order problems in [10,11,15]. This wide range of applications motivates us to make a contribution to this promising field.

The theory of fractional-order systems can be broadly divided into three subareas: the development of mathematical models, the analytical study of solutions, and the construction of computational schemes. The analytical aspect involves investigating the existence and uniqueness of solutions, bounds and periodicity, multiplicity of solutions, and the emergence of chaos. Several works have addressed the existence and uniqueness of solutions for nonlinear fractional-order partial differential equations (PDEs). Ouyang *et al*. [16] study such properties for PDEs with delay, while Rui *et al*. [17] explore the dynamics and existence of solutions via a dynamical system approach. Bonforte *et al*. [18] provide an optimal theory for the fractional heat equation. The boundedness of fractional operators is investigated in [19–21] by Das, Santra, and Cardoso *et al*., respectively. The persistence of multiplicity in fractional eigenvalue problems is studied by Ghimenti *et al*. [22], Rodrigues *et al*. [23], and Liang *et al*. [24] for fractional p-Laplace Choquard-Kirchhoff equations.

The development of computational methods for fractional-order systems is relatively challenging due to the complexity of fractional order operators and the large-scale calculations involved. Various approaches have been used to solve fractional-order systems, like transformation techniques such as the Laplace transform, Samudu transform, and natural transform. Numerical discretization methods and spectral methods are commonly used to solve these systems. Vivas-Cruz *et al*. in [25] introduce a hybrid finite element and Laplace transform method for efficient numerical solutions of fractional PDEs on graphics processing units. Firouzjaei *et al*. in [26] apply the Laplace transform and local discontinuous Galerkin methods to solve fourth-order time-fractional partial integro-differential equations with weakly singular kernels. The natural transform method is explored by Alsaud *et al*. in [27] to solve fractional coupled Burgers’ equations. Numerical discretization techniques are presented by Lee *et al*. in [28] for nonlinear fractional-order differential equations. Spectral methods are also discussed by Hafeez and Krawczuk in [29], while Ali in [30] investigates the application of the Chebyshev spectral method to a biological population model. Some other methods like differential transforation methods [39–42], numerical discrete method [43–46] are also used for the solution of fractional order systems. Interested readers can find useful information about spectral method in [47–53].

The development of computational methods for fractional-order systems is relatively challenging due to the complexity of fractional-order operators and the large-scale computations involved. Various techniques have been applied to solve these systems, including transformation methods such as the Laplace, Samudu, and natural transforms. Numerical discretization and spectral methods are also commonly employed. Vivas-Cruz *et al*. [25] propose a hybrid finite element and Laplace transform method for efficiently solving fractional PDEs on GPUs. Firouzjaei *et al*. [26] apply the Laplace transform with local discontinuous Galerkin methods to fourth-order time-fractional integro-differential equations with weakly singular kernels. Alsaud *et al*. [27] utilize the natural transform method for solving fractional coupled Burgers’ equations. Numerical discretization approaches are presented by Lee *et al*. [28], while spectral methods are discussed by Hafeez and Krawczuk [29], and Ali [30], who uses the Chebyshev spectral method for a biological population model. Other approaches include differential transformation methods [39–42] and numerical discrete techniques [43–46]. Readers interested in spectral methods can refer to [47–53] for further insights.

This work is a small step forward in the development of computational scheme for solution of fractional order systems. Our approaches is a spectral operational matrix method for the solution of fractional order system. Operational matrix techniques have become an essential approach for solving PDEs due to their efficiency and accuracy. Kumar *et al*. [31] introduced a collocation method using the operational matrix of fractional-order Lagrange polynomials, offering an effective way to handle space-time fractional PDEs. Talib *et al*. [32] developed a generalized operational matrix for mixed partial derivatives, providing solutions to multi-order fractional PDEs. Ray *et al*. [33] explored a two-dimensional wavelet-based operational matrix method to address variable-order fractional integro-differential equations. Khalil *et al*. [34] extended these techniques to nonlinear systems governed by Caputo fractional differential equations with integral-type boundary conditions. Singh *et al*. [35] applied an operational matrix method to tackle nonlinear reaction–advection–diffusion equations. In another study, Singh and Saha Ray [36] focused on stochastic fractional differential equations using the Lerch operational matrix method. Chaudhary *et al*. [37] demonstrated the ’effectiveness of the Vieta-Lucas operational matrix for solving fractional differential equation systems, while Enadi and Al-Jawary [38] addressed elliptic PDEs with mixed boundary conditions through operational matrices.

In a recent work [7], the author have developed a spectral method for the solution of the following fractional order two point nonlocal boundary value problem given as


$$\frac{\partial^{\sigma }u(x,y)}{\partial x^{\sigma }}+\frac{\partial^{\sigma }u(x,y)}{\partial y^{\sigma }}=-f.$$

We extend the same operational matrix method to solve a more generalized class of partial differential equations as given below.


$$\frac{\partial^{\alpha }X(x,t)}{\partial y^{\alpha }}\&=\lambda_{1}\frac{\partial^{\beta }X(x,t)}{\partial x^{\beta }}+\lambda_{2}\frac{\partial^{\beta }Y(x,t)}{\partial x^{\beta }}+\Theta_{1}(x,t)X(x,t)+\Theta_{2}(x,t)Y(x,t)+f(x,t),\;\frac{\partial^{\alpha }Y(x,t)}{\partial y^{\alpha }}\&=\lambda_{3}\frac{\partial^{\beta }X(x,t)}{\partial x^{\beta }}+\lambda_{4}\frac{\partial^{\beta }Y(x,t)}{\partial x^{\beta }}+\Theta_{3}(x,t)X(x,t)+\Theta_{4}(x,t)Y(x,t)+g(x,t),\;$$

with the following initial and two point boundary conditions


$$X(x,0)=f_{1}(x),\;X(0,t)=\kappa_{1}X(\zeta_{1},t)+g_{1}(t),\;X^{\prime }(x,0)=f_{2}(x),\;X(1,t)=\kappa_{2}X(\zeta_{2},t)+g_{2}(t),\;Y(x,0)=f_{3}(x),\;Y(0,t)=\kappa_{1}Y(\zeta_{1},t)+g_{3}(t),\;Y^{\prime }(x,0)=f_{4}(x),\;Y(1,t)=\kappa_{2}Y(\zeta_{2},t)+g_{4}(t).\;$$

Where α,β∈(1,2], $\zeta_{i}\in [0,1]$,$\kappa_{i}\in R$ and *f*_*i*_(*x*) and *g*_*i*_(*t*) are sufficiently known functions. α and β are the order of derivatives. The objective is to find a smooth approximation *X*(*x*,*t*) and *Y*(*x*,*t*) which satisfy both the pdes along with the two point boundary conditions. In [9], the authors used Legendre polynomials for finding the approximate solution of the linear part of the above system only under the influence of the initial conditions. This work extend the same method to handle the variable coefficients together with the nonlocal nature of the boundary conditions.

The method presented in this paper is based on the most simplest Legendre polynomials. New operational matrices are developed for these polynomails, which effectively transform the fractional-order system into a set of easily solvable Sylvester type algebraic equations. The scheme remains still very applicable even when the constants $\lambda_{i}$ are treated as variables.

The structure of the paper is as follows: In Sect 2 we gathers all the preliminary results which are essential for our analysis and method development. In Sect 3, we introduce newly constructed operational matrices and outline the key mathematical tools and concepts related to them. Sect 4 focuses on applying these matrices to develop a new numerical scheme for solving the problem on hand. In Sect 5, the proposed algorithm is validated through several test problems. Sect 6 provides a detailed discussion of the results. The paper ends with a detailed conclusion and future work suggestions.

## 1 Preliminaries

The following definition from fractional calculus is of our basic interest.

**Definition 1.1.** *Consider a function $\phi \in L^{1}([a,b],\mathbb{R})$ defined on the domain [a,b]⊂ℝ. For μ>0, the Riemann-Liouville fractional integral operator is expressed as*

$$I_{a+}^{\mu }\phi (x)=\frac{1}{\Gamma (\mu )}\int_{a}^{x}(x-\xi {)}^{\mu -1}\phi (\xi )\;d\xi ,$$ (1)

*where the integral is assumed to converge.*

**Definition 1.2.** *Let $\phi (x)\in C^{n}[a,b]$. The Caputo fractional derivative of order ν is given by*

$$D^{\nu }\phi (x)=\frac{1}{\Gamma (n-\nu )}\int_{a}^{x}\frac{\phi^{\left(n\right)}(\xi )}{(x-\xi {)}^{\nu +1-n}}\;d\xi ,\;n-1\leq \nu <n,\;n\in {\mathbb{Z}}_{+},$$ (2)

*provided the integral exists pointwise over (a,∞), where n=⌊ν⌋+1.*

Using (1) and (2), the following properties can be derived.

$$D^{\nu }x^{p}=\frac{\Gamma (1+p)}{\Gamma (1+p-\nu )}x^{p-\nu },\;I^{\mu }x^{p}=\frac{\Gamma (1+p)}{\Gamma (1+p+\mu )}x^{p+\mu },\;D^{\nu }k=0,$$ (3)

where *k* is any constant.

### 1.1 Function approximation and orthogonal polynomial systems

Let ℱ([0,1]) denote the space of continuous functions on the unit interval. For any f∈ℱ([0,1]), there exist multiple sequences of polynomials that converge uniformly to *f*. We focus on a particular sequence constructed using orthogonal polynomials (the Legendre polynomials). The fundamental Legendre polynomials $\{Q_{s}(t){\}}_{s=0}^{\infty }$ defined on [–1,1] satisfy the three-term recurrence relation (see S1 Code for implementation)

$$Q_{s+1}(t)=\left(\frac{2s+1}{s+1}\right)tQ_{s}(t)-\left(\frac{s}{s+1}\right)Q_{s-1}(t),\;Q_{0}(t)=1,\;Q_{1}(t)=t,\;s\in \mathbb{N}$$ (4)

To adapt these polynomials to the unit interval [0,1], we employ the linear transformation $x=\frac{t+1}{2}$. The resulting modified polynomials $\{Q_{s}(x){\}}_{s=0}^{\infty }$ can be expressed as

$$Q_{s}(x)=\sum_{j=0}^{s}\alpha_{s,j}x^{j},\;s\in {\mathbb{N}}_{0}.$$ (5)

Here the coefficients are given by

$$\alpha_{s,j}=(-1{)}^{s+j}\frac{(s+j)!}{(s-j)!(j!{)}^{2}}$$ (6)

A fundamental property of these polynomials is their orthogonality relation, given in the following relation.

$$\int_{0}^{1}Q_{m}(x)Q_{s}(x)dx=\left\{ \begin{array}{cc} 0, & m\neq s\\ \frac{1}{2s+1}, & m=s \end{array}\right.$$ (7)

This orthogonality enables function expansion. For h∈ℱ([0,1]), we can construct the *S*-term approximation:

$$h(x)\approx \sum_{s=0}^{S}\beta_{s}Q_{s}(x)$$ (8)

The coefficients in the above equation can be easily calculated, by multiplying the above equation with *Q*_*s*_(*x*) and using the orthogonality condition (7) to get (see S3 Code for computational details)

$$\beta_{s}=(2s+1)\int_{0}^{1}\#x2009;h(x)Q_{s}(x)dx$$ (9)

In matrix notation we can express it as

$$h(x)\approx {𝐁}_{S}^{T}\Phi_{S}(x)$$ (10)

where

$$\Phi_{S}(x)=[Q_{0}(x),Q_{1}(x),...,Q_{S}(x){]}^{T}$$ (11)

and

$${𝐁}_{S}^{T}=[\beta_{0},\beta_{1},...,\beta_{S}{]}^{T}$$ (12)

For two variable functions $h\in ℱ([0,1{]}^{2})$, the approximation can be extended to (see S2 Code for two-dimensional implementation)

$$h(x,t)\approx \sum_{k=1}^{S^{2}}\gamma_{k}\Psi_{k}(x,t)$$ (13)

where

$$\Psi_{k}(x,t)=Q_{i}(x)Q_{j}(t),\;k=Si+j+1$$ (14)

The bivariate orthogonality condition becomes

$$\int_{0}^{1}\int_{0}^{1}\Psi_{k}(x,t)\Psi_{m}(x,t)dxdt=\int_{0}^{1}\int_{0}^{1}Q_{i}(x)Q_{j}(t)Q_{a}(x)Q_{b}(t)dxdt\;=\frac{\delta_{j,b}\delta_{i,a}}{\left(2i+1)(2j+1\right)},\;k=Si+j+1,\;m=Sa+b+1$$ (15)

$\delta_{i,a}$ is the kronecker delta function. The coefficients are determined by

$$\gamma_{k}=(2i+1)(2j+1)\int_{0}^{1}\int_{0}^{1}\#x2009;h(x,t)Q_{i}(x)Q_{j}(t)dxdt$$ (16)

In compact form:

$$h(x,t)\approx \gamma_{S^{2}}^{T}\Psi_{S^{2}}(x,t)$$ (17)

where $\Psi_{S^{2}}$ is an $\left(S^{2}\times 1\right)$ vector and $\gamma_{S^{2}}^{T}$ is a $\left(1\times S^{2}\right)$ coefficient vector.

## 2 Development of operational matrices

In this section we will present some important results and proof for the construction of operational matrices. We will start with the results regarding the Legendre tripple product integration constant.

**Lemma 2.1.** *For the shifted Legendre polynomials, the triple product integral on the unit interval is given by the following relation.*

$$\int_{0}^{1}Q_{p}(x)Q_{q}(x)Q_{r}(x)dx={ℶ}^{\left(p,q,r\right)},$$ (18)

*where*

$${ℶ}^{\left(p,q,r\right)}=\sum_{i=0}^{p}\sum_{j=0}^{q}\sum_{k=0}^{r}\alpha_{\left(p,i\right)}\alpha_{\left(q,j\right)}\alpha_{\left(r,k\right)}\Theta_{\left(i,j,k\right)},$$ (19)

*with $\alpha_{\left(.,.\right)}$ defined in (6)*


$$\Theta_{\left(i,j,k\right)}=\frac{1}{\left(i+j+k+1\right)}.$$

**Theorem 2.1.** *For a function vector $\Psi_{S}(x,t)$, the fractional integral of order μ with respect to x is given by (see* S6 Code *for single dimension and* S7 Code *for two dimensions):*


$$I_{x}^{\mu }\Psi_{S}(x,t)=J_{x}^{\mu }\Psi_{S}(x,t),$$

*where $J_{x}^{\mu }$ is an $\left(S^{2}\times S^{2}\right)$ matrix*

$$J_{x}^{\mu }=(\begin{array}{cccccc} m_{1,1} & m_{1,2} & \cdots & m_{1,s} & \cdots & m_{1,S^{2}}\\ m_{2,1} & m_{2,2} & \cdots & m_{2,s} & \cdots & m_{2,S^{2}}\\ \vdots & \vdots & \ddots & \vdots & \vdots & \vdots \\ m_{u,1} & m_{u,2} & \cdots & m_{u,s} & \cdots & m_{u,S^{2}}\\ \vdots & \vdots & \vdots & \vdots & \ddots & \vdots \\ m_{S^{2},1} & m_{S^{2},2} & \cdots & m_{S^{2},s} & \cdots & m_{S^{2},S^{2}} \end{array})$$ (20)

*with elements defined as*


$$m_{u,s}=\sum_{z=0}^{i}\frac{(-1{)}^{i+z}(i+z)!}{\Gamma (z+\mu +1)z{!}^{2}(i-z)!}\sum_{s=1}^{S^{2}}\kappa_{s}.$$

$\kappa_{s}$ *are the expansion coefficients and defined as $\kappa_{s}=\delta_{j,d}(2c+1)\sum_{k=0}^{c}\frac{(-1{)}^{c+k}(c+k)!}{(z+\mu +c+1)(c-k)!k{!}^{2}}.$*

*Proof:* Consider the fractional integral of order μ for the general term of the function vector $\Psi_{S}(x,t)$ with respect to *x*


$$I_{x}^{\mu }\Psi_{u}(x,t)=I_{x}^{\mu }[Q_{i}(x)Q_{j}(t)]\;u=Si+j+1.$$

Using the definition of fractional integration, we can write the above equation as


$$I_{x}^{\mu }Q_{i}(x)Q_{j}(t)=\sum_{z=0}^{i}(-1{)}^{i+z}\frac{\Gamma (z+1)}{\Gamma (z+\mu +1)}\frac{(i+z)!}{z{!}^{2}(i-z)!}Q_{j}(t)x^{z+\mu }.$$

The term $x^{z+\mu }Q_{j}(t)$ can be expanded in terms of the orthogonal polynomials as follows.


$$x^{z+\mu }Q_{j}(t)=\sum_{c=0}^{S}\sum_{d=0}^{S}\kappa_{cd}Q_{c}(x)Q_{d}(t)=\sum_{s=1}^{S^{2}}\kappa_{s}Q_{c}(x)Q_{d}(t)\;s=Sc+d+1$$

where $\kappa_{s}$ are the expansion coefficients. The indices *c* and *d* can be connected with *s* in the same way as previous section. Using the orthogonality condition, we can determine $\kappa_{s}$


$$\kappa_{s}=(2c+1)(2d+1)\int_{0}^{1}\int_{0}^{1}Q_{j}(t)Q_{d}(t)Q_{c}(x)x^{z+\mu }dxdt,$$

which simplifies to


$$\kappa_{s}=(2c+1)\delta_{j,d}\int_{0}^{1}Q_{c}(x)x^{z+\mu }dx=\delta_{j,d}(2c+1)\sum_{k=0}^{c}\frac{(-1{)}^{c+k}(c+k)!}{(z+\mu +c+1)(c-k)!k{!}^{2}}.$$

Therefore


$$I_{x}^{\mu }Q_{i}(x)Q_{j}(t)=\sum_{z=0}^{i}(-1{)}^{i+z}\frac{\Gamma (z+1)}{\Gamma (z+\mu +1)}\frac{(i+z)!}{z{!}^{2}(i-z)!}\sum_{u=1}^{S^{2}}\kappa_{u}Q_{c}(x)Q_{d}(t).$$

Applying this for all i,j=0,1,2,…,S yields


$$I_{x}^{\mu }\Psi_{k}(x,t)=J_{x}^{\mu }\Psi_{k}(x,t),$$

where $J_{x}^{\mu }$ is the matrix representation of the fractional integral operator, and its elements are


$$m_{u,s}=\sum_{z=0}^{i}\frac{(-1{)}^{i+z}(i+z)!}{\Gamma (z+\mu +1)z{!}^{2}(i-z)!}\sum_{s=1}^{S^{2}}\kappa_{s}.$$

◻

**Theorem 2.2.** *For a function vector $\Psi_{S}(x,t)$, the fractional integral of order μ with respect to t is given by:*


$$J_{t}^{\mu }\Psi_{S}(x,t)=J_{t}^{\mu }\Psi_{S}(x,t),$$

*where $J_{t}^{\mu }$ is an $\left(S^{2}\times S^{2}\right)$ matrix*

$$J_{t}^{\mu }=(\begin{array}{cccccc} h_{1,1} & h_{1,2} & \cdots & h_{1,s} & \cdots & h_{1,S^{2}}\\ h_{2,1} & h_{2,2} & \cdots & h_{2,s} & \cdots & h_{2,S^{2}}\\ \vdots & \vdots & \ddots & \vdots & \vdots & \vdots \\ h_{u,1} & h_{u,2} & \cdots & h_{u,s} & \cdots & h_{u,S^{2}}\\ \vdots & \vdots & \vdots & \vdots & \ddots & \vdots \\ h_{S^{2},1} & h_{S^{2},2} & \cdots & h_{S^{2},s} & \cdots & h_{S^{2},S^{2}} \end{array})$$ (21)

*with elements defined as*


$$h_{u,s}=\sum_{z=0}^{j}\frac{(-1{)}^{j+z}(j+z)!}{\Gamma (z+\mu +1)z{!}^{2}(j-z)!}\sum_{s=1}^{S^{2}}\kappa_{s}.$$

$\kappa_{s}$ *are the expansion coefficients and defined as $\kappa_{s}=\delta_{i,c}(2d+1)\sum_{k=0}^{d}\frac{(-1{)}^{d+k}(d+k)!}{(z+\mu +c+1)(d-k)!k{!}^{2}}.$*

Proof: the proof of this Theorem is similar as the above Theorem ◻

**Theorem 2.3.** For a function vector $\Psi_{S}(x,t)$, the fractional derivative of order ν with respect to x is given by (see S4 Code for single dimension and S5 Code for matrix construction)


$$D_{x}^{\nu }\Psi_{S}(x,t)={𝐃}_{x}^{\nu }\Psi_{S}(x,t),$$

where ${𝐃}_{x}^{\nu }$ is an $\left(S^{2}\times S^{2}\right)$ matrix

$${𝐃}_{x}^{\nu }=(\begin{array}{cccccc} d_{1,1} & d_{1,2} & \cdots & d_{1,a} & \cdots & d_{1,S^{2}}\\ d_{2,1} & d_{2,2} & \cdots & d_{2,a} & \cdots & d_{2,S^{2}}\\ \vdots & \vdots & \ddots & \vdots & \vdots & \vdots \\ d_{u,1} & d_{u,2} & \cdots & d_{u,a} & \cdots & d_{u,S^{2}}\\ \vdots & \vdots & \vdots & \vdots & \ddots & \vdots \\ d_{S^{2},1} & d_{S^{2},2} & \cdots & d_{S^{2},a} & \cdots & d_{S^{2},S^{2}} \end{array}).$$ (22)

*The elements d_u,a_ are defined as*


$$d_{u,s}=\sum_{z=0}^{i}\sum_{a=1}^{S^{2}}(-1{)}^{z+i}\frac{\Gamma (1+z)(z+i)!}{\Gamma (1+z-\nu )(z!{)}^{2}(i-z)!}\eta_{a}.$$

*Proof:* Consider the fractional derivative of order ν for the function vector $\Psi_{S}(x,t)$ with respect to *x*


$$D_{x}^{\nu }Q_{i}(x)Q_{j}(t)=\sum_{z=0}^{i}(-1{)}^{i+z}\frac{\Gamma (1+z)(z+i)!}{\Gamma (1+z-\nu )(z!{)}^{2}(i-z)!}x^{z-\nu }Q_{j}(t).$$

The term $x^{z-\nu }Q_{j}(t)$ can be expressed using the orthogonal polynomials


$$x^{z-\nu }Q_{j}(t)=\sum_{c=0}^{S}\sum_{d=0}^{S}\eta_{cd}Q_{c}(x)Q_{d}(t)=\sum_{a=1}^{S^{2}}\eta_{a}Q_{c}(x)Q_{d}(t),$$

where $\eta_{a}$ are the expansion coefficients determined by the following relation.


$$\eta_{a}=(2c+1)(2d+1)\int_{0}^{1}\int_{0}^{1}Q_{j}(t)Q_{d}(t)Q_{c}(x)x^{z-\nu }dxdt.$$

This simplifies to


$$\eta_{a}=(2c+1)\delta_{j,d}\sum_{k=0}^{c}(-1{)}^{k+c}\frac{(k+c)!}{(z-\nu +k+1)(c-k)!(k!{)}^{2}}.$$

Therefore


$$D_{x}^{\nu }Q_{i}(x)Q_{j}(t)=\sum_{z=0}^{i}\sum_{a=1}^{S^{2}}(-1{)}^{i+z}\frac{\Gamma (1+z)(z+i)!}{\Gamma (1+z-\nu )(z!{)}^{2}(i-z)!}\eta_{a}Q_{c}(x)Q_{d}(t).$$

By applying this for all i,j=0,1,2,…,S, we obtain


$$D_{x}^{\nu }\Psi_{S}(x,t)={𝐃}_{x}^{\nu }\Psi_{S}(x,t),$$

where ${𝐃}_{x}^{\nu }$ represents the matrix form of the fractional derivative operator. This completes the proof. ◻

**Theorem 2.4.** *Let f(x,t) be any function in the set C([0,1]×[0,1]), and u(x,t) the unknown solution. Then (see* S8 Code *for variable coefficient implementation)*


$$f(x,t)u(x,t)=\gamma_{S^{2}}^{T}{𝔾}_{\left(S^{2}\times S^{2}\right)}^{f}\Psi_{S^{2}}(x,t),$$

*where $\gamma_{S^{2}}^{T}$ is the coefficient vector of u*(*x*,*t*), and

$$G_{\left(M^{2}\times S^{2}\right)}^{f}=(\begin{array}{cccccc} A_{\left(1,1\right)} & A_{\left(1,2\right)} & \cdots & A_{\left(1,s\right)} & \cdots & A_{\left(1,S^{2}\right)}\\ A_{\left(2,1\right)} & A_{\left(2,2\right)} & \cdots & A_{\left(2,s\right)} & \cdots & A_{\left(2,S^{2}\right)}\\ \vdots & \vdots & \ddots & \vdots & \vdots & \vdots \\ A_{\left(r\mathrm{.1}\right)} & A_{\left(r,2\right)} & \cdots & A_{\left(r,s\right)} & \cdots & A_{\left(r,S^{2}\right)}\\ \vdots & \vdots & \vdots & \vdots & \ddots & \vdots \\ A_{\left(S^{2},1\right)} & A_{\left(S^{2},2\right)} & \cdots & A_{\left(S^{2},s\right)} & \cdots & A_{\left(S^{2},S^{2}\right)} \end{array})$$ (23)

*where*


$$A_{\left(r,s\right)}=(2p+1)(2q+1)\sum_{n=1}^{S^{2}}c_{n}{ℶ}^{\left(a,i,p\right)}{ℷ}^{\left(a,i,p\right)}$$

*where the value of ${ℶ}^{\left(a,i,p\right)}$ and ${ℷ}^{\left(b,j,q\right)}$ is defined in Eq (19).*

*Proof:* Consider $u(x,t)≃\gamma_{S^{2}}^{T}\Psi_{S^{2}}(x,t{)}^{T}$, then we can write


$$f(x,t)u(x,t)=\gamma_{S^{2}}^{T}\overbrace{\Psi_{S^{2}}(x,t)}.$$

Where


$$\overbrace{\Psi_{S^{2}}(x,t)}=[\begin{array}{cccccc} X_{1}(x,t) & X_{2}(x,t) & \ldots & X_{r}(x,t & \ldots & X_{S^{2}}(x,t) \end{array}]T),$$

and


$$X_{r}(x,t)=f(x,t)\Psi_{r}(x,t).\;r=Sa+b+1\;\;fora,b=0,1,.....,s$$

The function *f*(*x*,*t*) can be expanded in ${\text{S}}^{2}$-terms of Legendre polynomials as


$$f(x,t)=\sum_{n=1}^{S^{2}}c_{n}\Psi_{n}(x,t),\;n=Si+j+1\;\;fori,j=0,1,.....,s$$

and substituting this relation gives


$$X_{r}(x,t)=\sum_{n=1}^{S^{2}}c_{n}\Psi_{n}(x,t)\Psi_{r}(x,t).$$

Using ${\text{S}}^{2}$-term Legendre approximations for *X*_*r*_(*x*,*t*), we have


$$X_{r}(x,t)=\sum_{s=1}^{S^{2}}A_{\left(r,s\right)}\Psi_{s}(x,t),\;s=Sp+q+1\;\;forp,q=0,1,.....,s$$

where


$$A_{\left(r,s\right)}=\sum_{n=1}^{S^{2}}c_{n}(2p+1)(2q+1)\int_{0}^{1}\int_{0}^{1}\Psi_{n}(x,t)\Psi_{r}(x,t)\Psi_{s}(x,t)\;dx\;dt.$$

Using the orthogonal properties of Legendre polynomials, we write


$$\Psi_{n}(x,t)=Q_{i}(x)Q_{j}(t),\;\text{where }n=Si+j+1,\;\;i,j=0,1,2,\ldots ,s,\;\Psi_{r}(x,t)=Q_{i}(x)Q_{j}(t),\;\text{where }r=Sa+b+1,\;\;a,b=0,1,2,\ldots ,s,\;\Psi_{s}(x,t)=Q_{p}(x)Q_{q}(t),\;\text{where }s=Sp+q+1\;\;p,q=0,1,2,\ldots ,s.$$

Substituting, we get


$$A_{\left(r,s\right)}=\sum_{n=1}^{S^{2}}c_{n}(2p+1)(2q+1)\int_{0}^{1}\int_{0}^{1}Q_{a}(x)Q_{i}(x)Q_{p}(x)Q_{b}(t)Q_{j}(t)Q_{q}(t)\;dx\;dt,$$

which simplifies to


$$A_{\left(r,s\right)}=\sum_{n=1}^{S^{2}}c_{n}(2p+1)(2q+1){ℶ}^{\left(a,i,p\right)}{ℷ}^{\left(b,j,q\right)}.$$

simulating the procedure for $r=1,\ldots ,S^{2}$ and $s=1,\ldots ,S^{2}$, we get:


$$(\begin{array}{c} X_{1}(x,t)\\ X_{2}(x,t)\\ \vdots \\ X_{r}(x,t)\\ \vdots \\ X_{S^{2}}(x,t) \end{array})=(\begin{array}{cccccc} \Lambda_{\left(1,1\right)} & \Lambda_{\left(1,2\right)} & \cdots & \Lambda_{\left(1,s\right)} & \cdots & \Lambda_{\left(1,S^{2}\right)}\\ \Lambda_{\left(2,1\right)} & \Lambda_{\left(2,2\right)} & \cdots & \Lambda_{\left(2,s\right)} & \cdots & \Lambda_{\left(2,S^{2}\right)}\\ \vdots & \vdots & \ddots & \vdots & \vdots & \vdots \\ \Lambda_{\left(r,1\right)} & \Lambda_{\left(r,2\right)} & \cdots & \Lambda_{\left(r,s\right)} & \cdots & \Lambda_{\left(r,S^{2}\right)}\\ \vdots & \vdots & \vdots & \vdots & \ddots & \vdots \\ \Lambda_{\left(S^{2},1\right)} & \Lambda_{\left(S^{2},2\right)} & \cdots & \Lambda_{\left(S^{2},s\right)} & \cdots & \Lambda_{\left(S^{2},S^{2}\right)} \end{array}).(\begin{array}{c} Q_{1}(x,t)\\ Q_{2}(x,t)\\ \vdots \\ Q_{r}(x,t)\\ \vdots \\ Q_{S^{2}}(x,t) \end{array}).$$

Thus


$$\overbrace{\Psi_{S^{2}}(x,t)}={𝔾}_{\left(S^{2}\times S^{2}\right)}^{f}\Psi_{S^{2}}(x,t).$$

This completes the proof of the theorem. ◻

While dealing with the boundary condition some other important matrices will be necessary. In the following theorem we develop a matrix which is important to convert the nonlocal boundary condition to algebraic structure.

**Lemma 2.2.** *Let ${𝒫}_{n}(x)=\alpha x^{n}$ be a polynomial, $\Psi_{S^{2}}(x,t)$ be a function vector and η∈ℝ, then*


$$\alpha x^{n}\Psi_{S^{2}}(\eta ,t)={𝐀}^{\left(\alpha ,n,\eta \right)}\Psi_{S^{2}}(x,t)$$

*The matrix ${𝐀}^{\left(\alpha ,n,\eta \right)}$ is defined as*


$${𝐀}^{\left(\alpha ,n,\eta \right)}=\left(\begin{array}{cccc} \Omega_{\left(1,1\right)} & \Omega_{\left(1,2\right)} & \cdots & \Omega_{\left(1,S^{2}\right)}\\ \Omega_{\left(2,1\right)} & \Omega_{\left(2,2\right)} & \cdots & \Omega_{\left(2,S^{2}\right)}\\ \vdots & \vdots & \ddots & \vdots \\ \Omega_{\left(S^{2},1\right)} & \Omega_{\left(S^{2},2\right)} & \cdots & \Omega_{\left(S^{2},S^{2}\right)} \end{array}\right),$$

*where $\Omega_{\left(r,s\right)}$ corresponds to ${𝒯}_{\left(i,j,k,l\right)}$ with *s* = *Sl* + *k* + 1, *r* = *Si* + *j* + 1, for i,j,k,l=0,1,2,…,S,*


$${𝒯}_{\left(i,j,k,l\right)}=\sum_{m=0}^{i}\frac{(i+m)!(-1{)}^{i+m}}{(m!{)}^{2}(i-m)!}\alpha \eta^{m}\sum_{k=0}^{S}\sum_{l=0}^{S}\Lambda_{\left(k,l\right)}.$$

*Proof:* The general term can be expressed as


$${𝒫}_{n}(x)Q_{i}(\eta )Q_{j}(t)=\alpha \sum_{m=0}^{i}\alpha_{\left(i,m\right)}\eta^{m}x^{n}Q_{j}(t).$$

Using *S*-terms Legendre approximation


$$Q_{j}(t)x^{n}=\sum_{k=0}^{S}\sum_{l=0}^{S}\Lambda_{\left(k,l\right)}Q_{k}(x)Q_{l}(t),$$

where


$$\Lambda_{\left(k,l\right)}=(2k+1)(2l+1)\int_{0}^{1}\int_{0}^{1}x^{n}Q_{k}(x)Q_{l}(t)Q_{j}(t)dxdt.$$

By orthogonality properties


$$\Lambda_{\left(k,l\right)}=(2k+1)\delta_{\left(j,l\right)}\int_{0}^{1}\sum_{p=0}^{k}\frac{(-1{)}^{k+p}(k+p)!}{(k-p)!(p!{)}^{2}}x^{n+p}dx,$$

$$\Lambda_{\left(k,l\right)}=(2k+1)\delta_{\left(j,l\right)}\sum_{p=0}^{k}\frac{(-1{)}^{k+p}(k+p)!}{(n+p+1)(k-p)!(p!{)}^{2}}.$$

Therefore:


$$\alpha x^{n}\Psi_{S^{2}}(\eta ,t)=\sum_{m=0}^{i}\sum_{l=0}^{S}\sum_{k=0}^{S}\frac{(-1{)}^{i+m}(i+m)!\alpha \eta^{m}\Lambda_{\left(k,l\right)}}{(i-m)!(m!{)}^{2}}Q_{k}(x)Q_{l}(t).$$

The matrix representation follows from this expression. ◻

**Lemma 2.3.** *Let Ψ be a polynomial vector and α,η∈ℝ, then*


$$\alpha \Psi_{S^{2}}(\eta ,t)={𝐂}^{\left(\alpha ,\eta \right)}\Psi .$$

*where*


$${𝐂}^{\left(\alpha ,\eta \right)}=\left(\begin{array}{cccc} \Xi_{\left(1,1\right)} & \Xi_{\left(1,2\right)} & \cdots & \Xi_{\left(1,S^{2}\right)}\\ \Xi_{\left(2,1\right)} & \Xi_{\left(2,2\right)} & \cdots & \Xi_{\left(2,S^{2}\right)}\\ \vdots & \vdots & \ddots & \vdots \\ \Xi_{\left(S^{2},1\right)} & \Xi_{\left(S^{2},2\right)} & \cdots & \Xi_{\left(S^{2},S^{2}\right)} \end{array}\right),$$

*with $\Xi_{\left(r,s\right)}={ℬ}_{i,j,k,l}$ and *r* = *Si* + *j* + 1, *s* = *Sl* + *k* + 1, k,l,i,j=0,1,2,…,S, and*


$${ℬ}_{i,j,k,l}=\sum_{m=0}^{i}\sum_{k=0}^{S}\sum_{l=0}^{S}\frac{(i+m)!\alpha \eta^{m}(-1{)}^{i+m}}{(i-m)!(m!{)}^{2}}\Lambda_{\left(k,l\right)}.$$

*Proof:* The proof follows analogously to Lemma 2.2. ◻

## 3 Method of solution

The operational matrices developed in the previous section plays central role in the development of the approximation procedure for the solution of nonlocal fractional order boundary value problems. We will apply these matrices to three different classes of these systems.

### 3.1 Solution methodology for fractional order partial differential equations with simple initial conditions

Consider the fractional order partial differential equation having the form

$$\frac{\partial^{\alpha }X(x,t)}{\partial t^{\alpha }}=\lambda \frac{\partial^{\beta }X(x,t)}{\partial x^{\beta }}+\theta (x,t)X(x,t)+f(x,t),$$ (24)

with initial conditions


$$X(x,0)=f_{1}(x)\;X^{\prime }(x,0)=f_{2}(x).$$

We assume that the solution can be expressed in terms of Legendre polynomials, such that


$$\frac{\partial^{\alpha }X(x,t)}{\partial t^{\alpha }}=\gamma_{{\text{S}}^{2}}^{\text{T}}\Psi_{{\text{S}}^{2}}(x,t),$$

By integrating of order α with respect to t and using the operational matrix of integration, we obtain the following relationship

$$X(x,t)=\gamma_{{\text{S}}^{2}}^{\text{T}}J_{t}^{\alpha }\Psi_{{\text{S}}^{2}}(x,t)+c_{0}+c_{1}t,$$ (25)

Upon utilizing the initial conditions *X*(*x*,0) = *f*_1_(*x*) and $X^{\prime }(x,0)=f_{2}(x)$ we can determine the values of $c_{0}=f_{1}(x)$ and $c_{1}=f_{2}(x)$. By substituting these values of *c*_0_ and *c*_1_ into Eq 25, we obtain


$$X(x,t)=\gamma_{{\text{S}}^{2}}^{\text{T}}J_{t}^{\alpha }\Psi_{{\text{S}}^{2}}(x,t)+tf_{2}(x)+f_{1}(x),$$

Let approximate the function in S-terms Legendre polynomials as


$$f_{1}(x)+tf_{2}(x)=F_{1\;{\text{S}}^{2}}^{\text{T}}\Psi_{{\text{S}}^{2}}(x,t)\;and\;f(x,t)=S_{{\text{S}}^{2}}^{\text{T}}\Psi_{{\text{S}}^{2}}(x,t),$$

$$X(x,t)=\gamma_{{\text{S}}^{2}}^{\text{T}}J_{t}^{\alpha }\Psi_{{\text{S}}^{2}}(x,t)+F_{1\;{\text{S}}^{2}}^{\text{T}}\Psi_{{\text{S}}^{2}}(x,t).$$

Let suppose


$$J^{\prime }=\gamma_{{\text{S}}^{2}}^{\text{T}}J_{t}^{\alpha }+F_{1\;{\text{S}}^{2}}^{\text{T}}$$

then


$$X(x,t)=J^{\prime }\Psi_{{\text{S}}^{2}}(x,t).$$

In matrix form we can write as


$$\frac{\partial^{\beta }X(x,t)}{\partial x^{\beta }}=J^{\prime }\;\;D_{x}^{\beta }\Psi_{{\text{S}}^{2}}(x,t),$$

$$\theta (x,t)X(x,t)=J^{\prime }\;\;G_{S^{2}\times S^{2}}^{\theta }\Psi_{{\text{S}}^{2}}(x,t).$$

Substituting these estimates into Eq 24 yields the following result


$$\gamma_{{\text{S}}^{2}}^{\text{T}}\Psi_{{\text{S}}^{2}}(x,t)=[\lambda J^{\prime }\;D_{x}^{\beta }\Psi_{{\text{S}}^{2}}(x,t)+J^{\prime }\;G_{S^{2}\times S^{2}}^{\theta }+S_{{\text{S}}^{2}}^{\text{T}}]\Psi_{{\text{S}}^{2}}(x,t),$$

After canceling $\Psi_{{\text{S}}^{2}}(x,t)$ from both sides and substituting the value of $J^{\prime }$, we can then


$$\gamma_{{\text{S}}^{2}}^{\text{T}}=\lambda \gamma_{{\text{S}}^{2}}^{\text{T}}J_{t}^{\alpha }D_{x}^{\beta }+\lambda F_{1\;{\text{S}}^{2}}^{\text{T}}D_{x}^{\beta }+\gamma_{{\text{S}}^{2}}^{\text{T}}J_{t}^{\alpha }G_{S^{2}\times S^{2}}^{\theta }+F_{1\;{\text{S}}^{2}}^{\text{T}}G_{S^{2}\times S^{2}}^{\theta }+S_{{\text{S}}^{2}}^{\text{T}},$$

further simplification, we can write as

$$\gamma_{{\text{S}}^{2}}^{\text{T}}=\gamma_{{\text{S}}^{2}}^{\text{T}}[\lambda J_{t}^{\alpha }D_{x}^{\beta }+J_{t}^{\alpha }G_{S^{2}\times S^{2}}^{\theta }]+\lambda F_{1\;{\text{S}}^{2}}^{\text{T}}D_{x}^{\beta }+F_{1\;{\text{S}}^{2}}^{\text{T}}G_{S^{2}\times S^{2}}^{\theta }+S_{{\text{S}}^{2}}^{\text{T}}.$$ (26)

The above system of algabric equation is a famous system of Sylvester type matrix equation, and can easily be solved for the unknown $\gamma_{{\text{S}}^{2}}^{\text{T}}$, once we calculate the solution of the above system it can easily be utilized to construct the spectral solution of the problem. In the next subsection we consider the same problem, but this time with a constrain of two point nonlocal type.

### 3.2 Solution methodology for fractional order partial differential equations with two-point non-local boundary conditions

Consider the problem defined in 24, together with the following conditions.


$$X(x,0)=f_{1}(x),\;X^{\prime }(x,0)=f_{2}(x),\;X(0,t)=\kappa_{1}X(\zeta_{1},t)+g_{1}(y),\;X(1,t)=\kappa_{2}X(\zeta_{2},t)+g_{2}(t).\;$$

We assume that the solution of the above problem exists and can be expressed in terms of shifted Legendre polynomials, such that


$$\frac{\partial^{\alpha }X(x,t)}{\partial t^{\alpha }}=\gamma_{{\text{S}}^{2}}^{\text{T}}\Psi_{{\text{S}}^{2}}(x,t),$$

Perform integration of order α with respect to *y*, we get

$$X(x,t)=\gamma_{{\text{S}}^{2}}^{\text{T}}J_{t}^{\alpha }\Psi_{{\text{S}}^{2}}(x,t)+c_{0}+c_{1}t,$$ (27)

After utilizing the initial conditions *X*(*x*,0) = *f*_1_(*x*) and $X^{\prime }(x,0)=f_{2}(x)$, and using the values of *c*_0_ and *c*_1_ in (27)


$$X(x,t)=\gamma_{{\text{S}}^{2}}^{\text{T}}J_{t}^{\alpha }\Psi_{{\text{S}}^{2}}(x,t)+tf_{2}(x)+f_{1}(x).$$

The function $f_{1}(x)+tf_{2}(x)$ and the source term *f*(*x*,*t*) can be expanded in terms of Legndre series


$$f_{1}(x)+tf_{2}(x)=F_{1\;{\text{S}}^{2}}^{\text{T}}\Psi_{{\text{S}}^{2}}(x,t)$$

and


$$f(x,t)=S_{{\text{S}}^{2}}^{\text{T}}\Psi_{{\text{S}}^{2}}(x,t)$$

then using these values in the main model we get

$$X(x,t)=\gamma_{{\text{S}}^{2}}^{\text{T}}J_{t}^{\alpha }\Psi_{{\text{S}}^{2}}(x,t)+F_{1\;{\text{S}}^{2}}^{\text{T}}\Psi_{{\text{S}}^{2}}(x,t),$$ (28)

For simplicity of notation assume


$$J^{\prime }=\gamma_{{\text{S}}^{2}}^{\text{T}}J_{t}^{\alpha }+F_{1\;{\text{S}}^{2}}^{\text{T}},$$

which leads us to the following estimates.


$$X(x,t)=J^{\prime }\Psi_{{\text{S}}^{2}}(x,t).$$

In the view of previous lemma, and re arranging Eq 24, we can write


$$\lambda \frac{\partial^{\beta }X(x,t)}{\partial x^{\beta }}=[\gamma_{{\text{S}}^{2}}^{\text{T}}(I-J_{t}^{\alpha }G_{S^{2}\times S^{2}}^{\theta })-F_{1\;{\text{S}}^{2}}^{\text{T}}G_{S^{2}\times S^{2}}^{\theta }-S_{{\text{S}}^{2}}^{\text{T}}]\Psi_{{\text{S}}^{2}}(x,t)$$

Simplifying the above estimates using


$$U=\frac{1}{\lambda }[\gamma_{{\text{S}}^{2}}^{\text{T}}(I-J_{t}^{\alpha }G_{S^{2}\times S^{2}}^{\theta (x,t)})-F_{1\;{\text{S}}^{2}}^{\text{T}}G_{S^{2}\times S^{2}}^{\theta (x,t)}-S_{{\text{S}}^{2}}^{\text{T}}].$$

Where *I* is the identity matrix of order $\left(S^{2}\times S^{2}\right)$


$$\frac{\partial^{\beta }X(x,t)}{\partial x^{\beta }}=U\Psi_{{\text{S}}^{2}}(x,t).$$

Performing an integration of order β with respect to the x

$$X(x,t)=UJ_{x}^{\beta }\Psi_{{\text{S}}^{2}}(x,t)+d_{0}+d_{1}x.$$ (29)

By specifying the boundary conditions, we obtain the following result.


$$X(0,t)=UJ_{x}^{\beta }\Psi_{{\text{S}}^{2}}(0,t)+d_{0},$$

The boundary conditon $\kappa_{1}X(\zeta_{1},t)+g_{1}(t)$ implies that


$$d_{0}=UJ_{x}^{\beta }\kappa_{1}\Psi_{{\text{S}}^{2}}(\zeta_{1},t)+\kappa_{1}d_{0}+\kappa_{1}\zeta_{1}d_{1}+g_{1}(t).$$

Solving for *d*_1_ let suppose $e_{0}=\frac{\left(\kappa_{1}-1\right)}{\kappa_{1}\zeta_{1}}$, $e_{1}=\frac{1}{\kappa_{1}\zeta_{1}}$, and $e_{2}=\frac{1}{\zeta_{1}}.$

$$d_{1}+e_{0}d_{0}=-e_{2}UJ_{x}^{\beta }\Psi_{{\text{S}}^{2}}(\zeta_{1},y)-e_{1}g_{1}(t).$$ (30)

By imposing the additional boundary condition $X(1,t)=\kappa_{2}X(\zeta_{2},t)+g_{2}(t)$ and solving for *d*_1_ and *d*_0_, we can further simplify the expression as follows


$$UJ_{x}^{\beta }\Psi_{{\text{S}}^{2}}(1,t)+d_{0}+d_{1}=UJ_{x}^{\beta }\kappa_{2}\Psi_{{\text{S}}^{2}}(\zeta_{2},t)+\kappa_{2}d_{0}+\kappa_{2}\zeta_{2}d_{1}+g_{2}(t).$$

$$d_{1}+\frac{\left(\kappa_{2}-1\right)}{\left(\kappa_{2}\zeta_{2}-1\right)}d_{0}=U\frac{1}{\left(\kappa_{2}\zeta_{2}-1\right)}J_{x}^{\beta }\Psi_{{\text{S}}^{2}}(1,t)-U\frac{\kappa_{2}}{\left(\kappa_{2}\zeta_{2}-1\right)}\Psi_{{\text{S}}^{2}}(\zeta_{2},t)\;-\frac{1}{\left(\kappa_{2}\zeta_{2}-1\right)}g_{2}(t).\;$$

For simplification, let $e_{3}=\frac{\left(\kappa_{2}-1\right)}{\left(\kappa_{2}\zeta_{2}-1\right)}$, $e_{4}=\frac{1}{\left(\kappa_{2}\zeta_{2}-1\right)}$, and $e_{5}=\frac{\kappa_{2}}{\left(\kappa_{2}\zeta_{2}-1\right)}$

$$d_{1}+e_{3}d_{0}=UJ_{x}^{\beta }e_{4}\Psi_{{\text{S}}^{2}}(1,y)-UJ_{x}^{\beta }e_{5}\Psi_{{\text{S}}^{2}}(\zeta_{2},t)-e_{4}g_{2}(t).$$ (31)

Subtracting (31) from (30) and solving for the value of *d*_0_, we obtain

$$d_{0}=-U\frac{e_{2}}{\left(e_{0}-e_{3}\right)}J_{x}^{\beta }\Psi_{{\text{S}}^{2}}(\zeta_{1},t)-U\frac{e_{4}}{\left(e_{0}-e_{3}\right)}J_{x}^{\beta }\Psi_{{\text{S}}^{2}}(1,t)\;+U\frac{e_{5}}{\left(e_{0}-e_{3}\right)}J_{x}^{\beta }\Psi_{{\text{S}}^{2}}(\zeta_{2},t)-\frac{e_{1}}{\left(e_{0}-e_{3}\right)}g_{1}(t)+\frac{e_{4}}{\left(e_{0}-e_{3}\right)}g_{2}(t).\;$$ (32)

further simplification of notations, w= let $a_{1}=\frac{e_{1}}{\left(e_{0}-e_{3}\right)}$, $a_{2}=\frac{e_{2}}{\left(e_{0}-e_{3}\right)}$, $a_{3}=\frac{e_{4}}{\left(e_{0}-e_{3}\right)}$, $a_{4}=\frac{e_{5}}{\left(e_{0}-e_{3}\right)}$

$$d_{0}=-a_{2}UJ_{x}^{\beta }\Psi_{{\text{S}}^{2}}(\zeta_{1},t)-a_{3}UJ_{x}^{\beta }\Psi_{{\text{S}}^{2}}(1,t)+a_{4}UJ_{x}^{\beta }\Psi_{{\text{S}}^{2}}(\zeta_{2},t)\;-a_{1}g_{1}(t)+a_{3}g_{2}(t).$$ (33)

Substituting the value of *d*_0_ obtained from the previous step into Eq 30 and solving for *d*_1_, we get

$$d_{1}=e_{0}a_{2}UJ_{x}^{\beta }\Psi_{{\text{S}}^{2}}(\zeta_{1},y)+e_{0}a_{3}UJ_{x}^{\beta }\Psi_{{\text{S}}^{2}}(1,t)-e_{0}a_{4}UJ_{x}^{\beta }\Psi_{{\text{S}}^{2}}(\zeta_{2},t)\;-e_{2}UJ_{x}^{\beta }\Psi_{{\text{S}}^{2}}(\zeta_{1},t)-e_{1}g_{1}(t)+e_{0}a_{1}g_{1}(t)-e_{0}a_{3}g_{2}(t).$$ (34)

After substituting the values of *d*_0_ and *d*_1_ in (29), we have

$$\gamma_{{\text{S}}^{2}}^{\text{T}}J_{t}^{\alpha }\Psi_{{\text{S}}^{2}}(x,t)=UJ_{x}^{\beta }\Psi_{{\text{S}}^{2}}(x,t)-a_{2}UJ_{x}^{\beta }\Psi_{{\text{S}}^{2}}(\zeta_{1},t)\;[6pt]-a_{3}UJ_{x}^{\beta }\Psi_{{\text{S}}^{2}}(1,t)+a_{4}UJ_{x}^{\beta }\Psi_{{\text{S}}^{2}}(\zeta_{2},t)\;[6pt]+e_{0}a_{2}UJ_{x}^{\beta }x\Psi_{{\text{S}}^{2}}(\zeta_{1},t)+e_{0}a_{3}UJ_{x}^{\beta }x\Psi_{{\text{S}}^{2}}(1,t)\;[6pt]-e_{0}a_{4}Ux\Psi_{{\text{S}}^{2}}(\zeta_{2},t)-e_{2}UJ_{x}^{\beta }x\Psi_{{\text{S}}^{2}}(\zeta_{1},t)-F_{1\;{\text{S}}^{2}}^{\text{T}}\Psi_{{\text{S}}^{2}}(x,t)\;[6pt]-e_{1}xg_{1}(t)-a_{1}g_{1}(t)+a_{3}g_{2}(t)+e_{0}a_{1}xg_{1}(t)-e_{0}a_{3}xg_{2}(t),\;$$ (35)

The function $-e_{1}xg_{1}(t)-a_{1}g_{1}(t)+a_{3}g_{2}(t)+e_{0}a_{1}xg_{1}(t)-e_{0}a_{3}xg_{2}(t)$ can be easily expanded in its series as follows,


$$F_{2\;{\text{S}}^{2}}^{\text{T}}\Psi_{{\text{S}}^{2}}(x,t)=-e_{1}xg_{1}(t)-a_{1}g_{1}(t)+a_{3}g_{2}(t)+e_{0}a_{1}xg_{1}(t)-e_{0}a_{3}xg_{2}(t).$$

We can write


$$cx^{n}\Psi_{{\text{S}}^{2}}(\zeta ,t)=A^{\left(c,n,\zeta \right)}\Psi_{{\text{S}}^{2}}(x,t),\;\;\;\;\;c\Psi_{{\text{S}}^{2}}(\zeta ,t)=C^{\left(c,\zeta \right)}\Psi_{{\text{S}}^{2}}(x,t).$$

$$\gamma_{{\text{S}}^{2}}^{\text{T}}J_{t}^{\alpha }=UJ_{x}^{\beta }-UJ_{x}^{\beta }C^{\left(a_{2},\zeta_{1}\right)}-UJ_{x}^{\beta }C^{\left(a_{3},1\right)}\;+UJ_{x}^{\beta }C^{\left(a_{4},\zeta_{2}\right)}+UJ_{x}^{\beta }A^{\left(e_{0}a_{2},1,\zeta_{1}\right)}+UJ_{x}^{\beta }A^{\left(e_{0}a_{3},1,1\right)}\;-UJ_{x}^{\beta }A^{\left(e_{0}a_{4},1,\zeta_{2}\right)}-UJ_{x}^{\beta }A^{\left(e_{2},1,\zeta_{1}\right)}+F_{2\;{\text{S}}^{2}}^{\text{T}}-F_{1\;{\text{S}}^{2}}^{\text{T}}.\;$$

$$\gamma_{{\text{S}}^{2}}^{\text{T}}J_{t}^{\alpha }=\gamma_{{\text{S}}^{2}}^{\text{T}}(II-J_{t}^{\alpha }G\theta_{2})J_{x}^{\beta }[II-C^{\left(a_{2},\zeta_{1}\right)}-C^{\left(a_{3},1\right)}+C^{\left(a_{4},\zeta_{2}\right)}\;+A^{\left(e_{0}a_{2},1,\zeta_{1}\right)}+A^{\left(e_{0}a_{3},1,1\right)}-A^{\left(e_{0}a_{4},1,\zeta_{2}\right)}-A^{\left(e_{2},1,\zeta_{1}\right)}]\;-[F_{1}(G^{\theta_{2}})-S]J_{x}^{\beta }[II-C^{\left(a_{2},\zeta \right)}-C^{\left(a_{3},1\right)}+C^{\left(a_{4},\zeta_{2}\right)}-A^{\left(e_{0}a_{2},1,\zeta_{1}\right)}+A^{\left(e_{0}a_{3},1,1\right)}\;-A^{\left(e_{0}a_{4},1,\zeta_{2}\right)}-A^{\left(e_{2},1,\zeta_{1}\right)}]+F_{2\;{\text{S}}^{2}}^{\text{T}}-F_{1\;{\text{S}}^{2}}^{\text{T}}.\;$$ (36)

The equations derived above have a desired algebraic structure, which can be solved efficiently using the Matlab platform to find the unknown $\gamma_{{\text{S}}^{2}}^{\text{T}}$ row vector. Once the value of $\gamma_{{\text{S}}^{2}}^{\text{T}}$ is determined, it can be substituted into \eqreft2pnomain to obtain an approximation of the solution to the problem. In the next subsection we are going to extend the theory for the coupled systems of fractional order nonlocal boundary conditions.

### 3.3 Solution methodology for the generalized coupled systems

Considered the coupled system of fractional order PDEs

$$\frac{\partial^{\alpha }X(x,t)}{\partial t^{\alpha }}=\lambda_{1}\frac{\partial^{\beta }X(x,t)}{\partial x^{\beta }}+\lambda_{2}\frac{\partial^{\beta }Y(x,t)}{\partial x^{\beta }}+\Theta_{1}(x,t)X(x,t)+\Theta_{2}(x,t)Y(x,t)+f(x,t),\;[8pt]\frac{\partial^{\alpha }Y(x,t)}{\partial t^{\alpha }}=\lambda_{3}\frac{\partial^{\beta }X(x,t)}{\partial x^{\beta }}+\lambda_{4}\frac{\partial^{\beta }Y(x,t)}{\partial x^{\beta }}+\Theta_{3}(x,t)X(x,t)+\Theta_{4}(x,t)Y(x,t)+g(x,t),\;$$ (37)

with the following initial and boundary conditions


$$X(x,0)=f_{1}(x),\;X(0,t)=\kappa_{1}X(\zeta_{1},t)+g_{1}(t),\;X^{\prime }(x,0)=f_{2}(x),\;X(1,t)=\kappa_{2}X(\zeta_{2},t)+g_{2}(t),\;Y(x,0)=f_{3}(x),\;Y(0,t)=\kappa_{1}Y(\zeta_{1},t)+g_{3}(t),\;Y^{\prime }(x,0)=f_{4}(x),\;Y(1,t)=\kappa_{2}Y(\zeta_{2},t)+g_{4}(t).\;$$

We assume that


$$\frac{\partial^{\alpha }X(x,t)}{\partial t^{\alpha }}=K_{{\text{S}}^{2}}^{\text{T}}\Psi_{{\text{S}}^{2}}(x,t),\;\frac{\partial^{\alpha }Y(x,t)}{\partial t^{\alpha }}=L_{{\text{S}}^{2}}^{\text{T}}\Psi_{{\text{S}}^{2}}(x,t).\;$$

By taking integration with respect to *t* of order α, and using the initial conditions, the S-terms Legendre approximation of


$$F_{1\;{\text{S}}^{2}}^{\text{T}}\Psi_{{\text{S}}^{2}}(x,t)=f_{1}(x)+tf_{2}(x),\;S_{1\;{\text{S}}^{2}}^{\text{T}}\Psi_{{\text{S}}^{2}}(x,t)=f(x,t),\;F_{2\;{\text{S}}^{2}}^{\text{T}}\Psi_{{\text{S}}^{2}}(x,t)=f_{3}(x)+tf_{4}(x),\;S_{2\;{\text{S}}^{2}}^{\text{T}}\Psi_{{\text{S}}^{2}}(x,t)=g(x,t),$$

we get the following results

$$X(x,t)=K_{{\text{S}}^{2}}^{\text{T}}J_{t}^{\alpha }\Psi_{{\text{S}}^{2}}(x,t)+F_{1\;{\text{S}}^{2}}^{\text{T}}\Psi_{{\text{S}}^{2}}(x,t)=K^{\prime }\Psi_{{\text{S}}^{2}}(x,t),$$ (38)

$$Y(x,t)=L_{{\text{S}}^{2}}^{\text{T}}J_{t}^{\alpha }\Psi_{{\text{S}}^{2}}(x,t)+F_{2\;{\text{S}}^{2}}^{\text{T}}\Psi_{{\text{S}}^{2}}(x,t)=L^{\prime }\Psi_{{\text{S}}^{2}}(x,t).$$

we follows


$$\lambda_{i}\frac{\partial^{\beta }Y(x,t)}{\partial x^{\beta }}=\lambda_{i}L^{\prime }D_{x}^{\beta }\Psi_{{\text{S}}^{2}}(x,t)\;\lambda_{j}\frac{\partial^{\beta }X(x,t)}{\partial x^{\beta }}=\lambda_{j}K^{\prime }D_{x}^{\beta }\Psi_{{\text{S}}^{2}}(x,t)\;\Theta_{i}(x,t)X(x,t)=K^{\prime }\;\;G^{\Theta_{i}}\Psi_{{\text{S}}^{2}}(x,t)\;\Theta_{j}(x,t)Y(x,t)=L^{\prime }\;\;G^{\Theta_{j}}\Psi_{{\text{S}}^{2}}(x,t)\;$$

By substituting these estimates into \eqrefcpsysmain and rearranging the terms, we obtain:


$$\lambda_{1}\frac{\partial^{\beta }X(x,t)}{\partial x^{\beta }}=[K_{{\text{S}}^{2}}^{\text{T}}-\lambda_{2}L^{\prime }D_{x}^{\beta }-L^{\prime }G^{\Theta_{2}}-K^{\prime }G^{\Theta_{1}}-S_{1}]\Psi_{{\text{S}}^{2}}(x,t)\;\lambda_{4}\frac{\partial^{\beta }Y(x,t)}{\partial x^{\beta }}=[L_{{\text{S}}^{2}}^{\text{T}}-\lambda_{3}K^{\prime }D_{x}^{\beta }-K^{\prime }G^{\Theta_{3}}-L^{\prime }G^{\Theta_{4}}-S_{2}]\;$$

Re-arranging the term and putting the values of $K^{\prime }$ and $L^{\prime }$. For the sake of simplicity, let suppose


$$W=\frac{1}{\lambda_{1}}[K_{{\text{S}}^{2}}^{\text{T}}(II-J_{t}^{\alpha }G^{\Theta_{1}})-L_{{\text{S}}^{2}}^{\text{T}}(\lambda_{2}J_{t}^{\alpha }D_{x}^{\beta }+J_{t}^{\alpha }G^{\theta_{2}})\;-F_{1}G^{\Theta_{1}}-F_{2}(\lambda_{2}D_{x}^{\beta }+G^{\Theta_{2}})-S_{1}],\;V=\frac{1}{\lambda_{4}}[L_{{\text{S}}^{2}}^{\text{T}}(II-J_{t}^{\alpha }G^{\Theta_{4}})-K_{{\text{S}}^{2}}^{\text{T}}(\lambda_{3}J_{t}^{\alpha }D_{x}^{\beta }+J_{t}^{\alpha }G^{\Theta_{3}})\;-F_{1}(\lambda_{3}D_{x}^{\beta }+G^{\Theta_{3}})-F_{2}G^{\Theta_{4}}-S_{2}].\;$$

Using the above notation and integrating with respect to *x* of order β

$$X(x,t)=WJ_{x}^{\beta }\Psi_{{\text{S}}^{2}}(x,t)+d_{0}+xd_{1},\;[4pt]Y(x,t)=VJ_{x}^{\beta }\Psi_{{\text{S}}^{2}}(x,t)+f_{0}+xf_{1}.\;$$ (39)

By imposing the boundary conditions $X(0,t)=\kappa_{1}X(\zeta_{1},t)+g_{1}(t)$ and $Y(0,t)=\kappa_{1}X(\zeta_{1},t)+g_{3}(t)$, we get the following estimates.


$$\kappa_{1}X(\zeta_{1},t)+g_{1}(t)=WJ_{x}^{\beta }\kappa_{1}\Psi_{{\text{S}}^{2}}(\zeta_{1},t)+\kappa_{1}d_{0}+\kappa_{1}\zeta_{1}d_{1}+g_{1}(t),\;[4pt]\kappa_{1}Y(\zeta_{1},t)+g_{3}(t)=VJ_{x}^{\beta }\kappa_{1}\Psi_{{\text{S}}^{2}}(\zeta_{1},t)+\kappa_{1}f_{0}+\kappa_{1}\zeta_{1}f_{1}+g_{3}(t).$$

$$d_{0}=WJ_{x}^{\beta }\kappa_{1}\Psi_{{\text{S}}^{2}}(\zeta_{1},t)+\kappa_{1}d_{0}+\kappa_{1}\zeta_{1}d_{1}+g_{1}(t),\;[4pt]f_{0}=VJ_{x}^{\beta }\kappa_{1}\Psi_{{\text{S}}^{2}}(\zeta_{1},t)+\kappa_{1}f_{0}+\kappa_{1}\zeta_{1}f_{1}+g_{3}(t).\;$$

$$d_{1}+\frac{\left(\kappa_{1}-1\right)}{\kappa_{1}\zeta_{1}}d_{0}=-W\frac{\kappa_{1}}{\kappa_{1}\zeta_{1}}J_{x}^{\beta }\Psi_{{\text{S}}^{2}}(\zeta_{1},t)-\frac{1}{\kappa_{1}\zeta_{1}}g_{1}(t),\;[4pt]f_{1}+\frac{\left(\kappa_{1}-1\right)}{\kappa_{1}\zeta_{1}}f_{0}=-V\frac{\kappa_{1}}{\kappa_{1}\zeta_{1}}J_{x}^{\beta }\Psi_{{\text{S}}^{2}}(\zeta_{1},t)-\frac{1}{\kappa_{1}\zeta_{1}}g_{3}(t).\;$$

Let $e_{0}=\frac{\left(\kappa_{1}-1\right)}{\kappa_{1}\zeta_{1}}$, $e_{1}=\frac{1}{\kappa_{1}\zeta_{1}}$, and $e_{2}=\frac{1}{\zeta_{1}}.$

$$d_{1}+e_{0}d_{0}=-We_{2}J_{x}^{\beta }\Psi_{{\text{S}}^{2}}(\zeta_{1},t)-e_{1}g_{1}(t),\;f_{1}+e_{0}f_{0}=-Ve_{2}J_{x}^{\beta }\Psi_{{\text{S}}^{2}}(\zeta_{1},t)-e_{1}g_{3}(t).\;$$ (40)

Similarly by putting the boundary conditions $X(1,t)=\kappa_{2}X(\zeta_{2},t)\;+\;g_{2}(t)$ and $Y(1,t)=\kappa_{2}Y(\zeta_{2},t)+g_{4}(t),$ we get


$$\kappa_{2}X(\zeta_{2},t)+g_{2}(t)=W\kappa_{2}J_{x}^{\beta }\Psi_{{\text{S}}^{2}}(\zeta_{2},t)+\kappa_{2}d_{0}+\kappa_{2}\zeta_{2}d_{1}+g_{2}(t),\;\kappa_{2}Y(\zeta_{2},t)+g_{4}(t)=V\kappa_{2}J_{x}^{\beta }\Psi_{{\text{S}}^{2}}(\zeta_{2},t)+\kappa_{2}f_{0}+\kappa_{2}\zeta_{2}f_{1}+g_{4}(t).\;$$

By combining the equations obtained from integrating with respect to *x*, we can solve for *d*_1_ and *d*_0_ and simplify the expression further as follows


$$WJ_{x}^{\beta }\Psi_{{\text{S}}^{2}}(1,t)+d_{0}+d_{1}=WJ_{x}^{\beta }\kappa_{2}\Psi_{{\text{S}}^{2}}(\zeta_{2},t)+\kappa_{2}d_{0}+\kappa_{2}\zeta_{2}d_{1}+g_{2}(t),\;VJ_{x}^{\beta }\Psi_{{\text{S}}^{2}}(1,t)+f_{0}+f_{1}=VJ_{x}^{\beta }\kappa_{2}\Psi_{{\text{S}}^{2}}(\zeta_{2},t)+\kappa_{2}f_{0}+\kappa_{2}\zeta_{2}f_{1}+g_{4}(t).\;$$

$$d_{1}+\frac{\left(\kappa_{2}-1\right)}{\left(\kappa_{2}\zeta_{2}-1\right)}d_{0}=W\frac{1}{\left(\kappa_{2}\zeta_{2}-1\right)}J_{x}^{\beta }\Psi_{{\text{S}}^{2}}(1,t)-W\frac{\kappa_{2}}{\left(\kappa_{2}\zeta_{2}-1\right)}\Psi_{{\text{S}}^{2}}(\zeta_{2},t)\;-\frac{1}{\left(\kappa_{2}\zeta_{2}-1\right)}g_{2}(t),\;f_{1}+\frac{\left(\kappa_{2}-1\right)}{\left(\kappa_{2}\zeta_{2}-1\right)}f_{0}=V\frac{1}{\left(\kappa_{2}\zeta_{2}-1\right)}J_{x}^{\beta }\Psi_{{\text{S}}^{2}}(1,t)-V\frac{\kappa_{2}}{\left(\kappa_{2}\zeta_{2}-1\right)}\Psi_{{\text{S}}^{2}}(\zeta_{2},t)\;-\frac{1}{\left(\kappa_{2}\zeta_{2}-1\right)}g_{4}(t),\;$$

Let $e_{3}=\frac{\left(\kappa_{2}-1\right)}{\left(\kappa_{2}\zeta_{2}-1\right)}$, $e_{4}=\frac{1}{\left(\kappa_{2}\zeta_{2}-1\right)}$, and $e_{5}=\frac{\kappa_{2}}{\left(\kappa_{2}\zeta_{2}-1\right)},$

$$d_{1}+e_{3}d_{0}=We_{4}J_{x}^{\beta }\Psi_{{\text{S}}^{2}}(1,t)-We_{5}{JJ}_{x}^{\beta }\Psi_{{\text{S}}^{2}}(\zeta_{2},t)-e_{4}g_{2}(t),\;f_{1}+e_{3}f_{0}=Ve_{4}J_{x}^{\beta }\Psi_{{\text{S}}^{2}}(1,t)-Ve_{5}J_{x}^{\beta }\Psi_{{\text{S}}^{2}}(\zeta_{2},t)-e_{4}g_{4}(t).\;$$ (41)

We can obtain the value of *d*_0_ by subtracting Eq 41 from Eq 40 and solving for it as follows


$$(e_{0}-e_{3})d_{0}=-e_{3}WJ_{x}^{\beta }\Psi_{{\text{S}}^{2}}(\zeta_{1},t)-We_{4}J_{x}^{\beta }\Psi_{{\text{S}}^{2}}(1,t)+e_{5}WJ_{x}^{\beta }\Psi_{{\text{S}}^{2}}(\zeta_{2},t)\;-e_{1}g_{1}(t)+e_{4}g_{2}(t),\;[3pt](e_{0}-e_{3})f_{0}=-e_{3}VJ_{x}^{\beta }\Psi_{{\text{S}}^{2}}(\zeta_{1},t)-e_{4}VJ_{x}^{\beta }\Psi_{{\text{S}}^{2}}(1,t)+e_{5}VJ_{x}^{\beta }\Psi_{{\text{S}}^{2}}(\zeta_{2},t)\;-e_{1}g_{3}(t)+e_{4}g_{4}(t).$$

$$d_{0}=-W\frac{e_{2}}{\left(e_{0}-e_{3}\right)}J_{x}^{\beta }\Psi_{{\text{S}}^{2}}(\zeta_{1},t)-W\frac{e_{4}}{\left(e_{0}-e_{3}\right)}J_{x}^{\beta }\Psi_{{\text{S}}^{2}}(1,t)\;+W\frac{e_{5}}{\left(e_{0}-e_{3}\right)}J_{x}^{\beta }\Psi_{{\text{S}}^{2}}(\zeta_{2},t)-\frac{e_{1}}{\left(e_{0}-e_{3}\right)}g_{1}(t)+\frac{e_{4}}{\left(e_{0}-e_{3}\right)}g_{2}(t),\;f_{0}=-V\frac{e_{2}}{\left(e_{0}-e_{3}\right)}J_{x}^{\beta }\Psi_{{\text{S}}^{2}}(\zeta_{1},t)-V\frac{e_{4}}{\left(e_{0}-e_{3}\right)}J_{x}^{\beta }\Psi_{{\text{S}}^{2}}(1,t)\;+V\frac{e_{5}}{\left(e_{0}-e_{3}\right)}J_{x}^{\beta }\Psi_{{\text{S}}^{2}}(\zeta_{2},t)-\frac{e_{1}}{\left(e_{0}-e_{3}\right)}g_{3}(t)+\frac{e_{4}}{\left(e_{0}-e_{3}\right)}g_{4}(t).\;$$ (42)

Let $a_{1}=\frac{e_{1}}{\left(e_{0}-e_{3}\right)}$, $a_{2}=\frac{e_{2}}{\left(e_{0}-e_{3}\right)}$, $a_{3}=\frac{e_{4}}{\left(e_{0}-e_{3}\right)}$, $a_{4}=\frac{e_{5}}{\left(e_{0}-e_{3}\right)}.$

$$d_{0}=-a_{2}WJ_{x}^{\beta }\Psi_{{\text{S}}^{2}}(\zeta_{1},t)-a_{3}WJ_{x}^{\beta }\Psi_{{\text{S}}^{2}}(1,t)+a_{4}J_{x}^{\beta }\Psi_{{\text{S}}^{2}}(\zeta_{2},t)\;-a_{1}g_{1}(t)+a_{3}g_{2}(t),\;f_{0}=-a_{2}VJ_{x}^{\beta }\Psi_{{\text{S}}^{2}}(\zeta_{1},t)-a_{3}VJ_{x}^{\beta }\Psi_{{\text{S}}^{2}}(1,t)+a_{4}VJ_{x}^{\beta }\Psi (\zeta_{2},t)\;-a_{1}g_{3}(t)+a_{3}g_{4}(t).$$ (43)

Now putting value of *d*_0_ in \eqrefeqa11, and solving for *d*_1_


$$d_{1}-e_{0}a_{2}WJ_{x}^{\beta }\Psi_{{\text{S}}^{2}}(\zeta_{1},t)-e_{0}a_{3}WJ_{x}^{\beta }\Psi_{{\text{S}}^{2}}(1,t)+We_{0}a_{4}J_{x}^{\beta }\Psi_{{\text{S}}^{2}}(\zeta_{2},t)\;-e_{0}a_{1}g_{1}(t)+e_{0}a_{3}g_{2}(t)=-e_{2}WJ_{x}^{\beta }\Psi_{{\text{S}}^{2}}(\zeta_{1},t)-e_{1}g_{1}(t).\;$$

Similarly putting value of *f*_0_ and solving for *f*_1_

$$f_{1}=Ve_{0}a_{2}J_{x}^{\beta }\Psi_{{\text{S}}^{2}}(\zeta_{1},t)+Ve_{0}a_{3}J_{x}^{\beta }\Psi_{{\text{S}}^{2}}(1,t)-Ve_{0}a_{4}J_{x}^{\beta }\Psi_{{\text{S}}^{2}}(\zeta_{2},t)\;-Ve_{2}J_{x}^{\beta }\Psi_{{\text{S}}^{2}}(\zeta_{1},t)-e_{1}g_{3}(t)+e_{0}a_{1}g_{3}(t)-e_{0}a_{3}g_{4}(t).\;$$ (44)

Using the vales of *d*_0_ and *d*_1_ we can write

$$k_{{\text{S}}^{2}}^{\text{T}}J_{t}^{\alpha }\Psi_{{\text{S}}^{2}}(x,t)=WJ_{x}^{\beta }\Psi_{{\text{S}}^{2}}(x,t)-Wa_{2}J_{x}^{\beta }\Psi_{{\text{S}}^{2}}(\zeta_{1},t)\;[2pt]-Wa_{3}J_{x}^{\beta }\Psi_{{\text{S}}^{2}}(1,t)+Wa_{4}J_{x}^{\beta }\Psi_{{\text{S}}^{2}}(\zeta_{2},t)\;[2pt]+We_{0}a_{2}J_{x}^{\beta }x\Psi_{{\text{S}}^{2}}(\zeta_{1},t)+We_{0}a_{3}J_{x}^{\beta }x\Psi_{{\text{S}}^{2}}(1,t)\;[2pt]-We_{0}a_{4}x\Psi_{{\text{S}}^{2}}(\zeta_{2},t)-We_{2}J_{x}^{\beta }x\Psi_{{\text{S}}^{2}}(\zeta_{1},t)\;-F_{1\;{\text{S}}^{2}}^{\text{T}}\Psi_{{\text{S}}^{2}}(x,t)-e_{1}xg_{1}(t)-a_{1}g_{1}(t)+a_{3}g_{2}(t)+e_{0}a_{1}xg_{1}(t)-e_{0}a_{3}xg_{2}(t).\;$$ (45)

Similarly putting the vales of *f*_0_ and *f*_1_ we can write

$$L_{{\text{S}}^{2}}^{\text{T}}J_{t}^{\alpha }\Psi_{{\text{S}}^{2}}(x,t)=VJ_{x}^{\beta }\Psi_{{\text{S}}^{2}}(x,t)-Va_{2}J_{x}^{\beta }\Psi_{{\text{S}}^{2}}(\zeta_{1},t)\;[4pt]-Va_{3}J_{x}^{\beta }\Psi_{{\text{S}}^{2}}(1,t)+Va_{4}J_{x}^{\beta }\Psi_{{\text{S}}^{2}}(\zeta_{2},t)\;[4pt]+Ve_{0}a_{2}J_{x}^{\beta }x\Psi_{{\text{S}}^{2}}(\zeta_{1},t)+Ve_{0}a_{3}J_{x}^{\beta }x\Psi_{{\text{S}}^{2}}(1,t)\;[4pt]-Ve_{0}a_{4}x\Psi (\zeta_{2},t)-Ve_{2}J_{x}^{\beta }x\Psi (\zeta_{1},t)\;[4pt]-F_{2\;{\text{S}}^{2}}^{\text{T}}\Psi_{{\text{S}}^{2}}(x,t)-e_{1}xg_{3}(t)-a_{1}g_{3}(t)+a_{3}g_{4}(t)+e_{0}a_{1}xg_{3}(t)-e_{0}a_{3}xg_{4}(t).$$ (46)

let


$$F_{3\;{\text{S}}^{2}}^{\text{T}}\Psi_{{\text{S}}^{2}}(x,t)=-e_{1}xg_{1}(t)-a_{1}g_{1}(t)+a_{3}g_{2}(t)+e_{0}a_{1}xg_{1}(t)-e_{0}a_{3}xg_{2}(y),$$

$$F_{4\;{\text{S}}^{2}}^{\text{T}}\Psi_{{\text{S}}^{2}}(x,t)=-e_{1}xg_{3}(t)-a_{1}g_{3}(t)+a_{3}g_{4}(t)+e_{0}a_{1}xg_{3}(t)-e_{0}a_{3}xg_{4}(t),$$

we can write

$$K_{{\text{S}}^{2}}^{\text{T}}J_{t}^{\alpha }=WJ_{x}^{\beta }-WJ_{x}^{\beta }C^{\left(a_{2},\zeta_{1}\right)}-WJ_{x}^{\beta }C^{\left(a_{3},1\right)}\;[4pt]+WJ_{x}^{\beta }C^{\left(a_{4},\zeta_{2}\right)}+WJ_{x}^{\beta }A^{\left(e_{0}a_{2},1,\zeta_{1}\right)}+WJ_{x}^{\beta }A^{\left(e_{0}a_{3},1,1\right)}\;[4pt]-WJ_{x}^{\beta }A^{\left(e_{0}a_{4},1,\zeta_{2}\right)}-WJ_{x}^{\beta }A^{\left(e_{2},1,\zeta_{1}\right)}+F_{3\;{\text{S}}^{2}}^{\text{T}}-F_{1\;{\text{S}}^{2}}^{\text{T}},\;[4pt]L_{{\text{S}}^{2}}^{\text{T}}J_{t}^{\alpha }=VJ_{x}^{\beta }-VJ_{x}^{\beta }C^{\left(a_{2},\zeta_{1}\right)}-VJ_{x}^{\beta }C^{\left(a_{3},1\right)}\;[4pt]+VJ_{x}^{\beta }C^{\left(a_{4},\zeta_{2}\right)}+VJ_{x}^{\beta }A^{\left(e_{0}a_{2},1,\zeta_{1}\right)}+VJ_{x}^{\beta }A^{\left(e_{0}a_{3},1,1\right)}\;-VJ_{x}^{\beta }A^{\left(e_{0}a_{4},1,\zeta_{2}\right)}-VJ_{x}^{\beta }A^{\left(e_{2},1,\zeta_{1}\right)}+F_{4\;{\text{S}}^{2}}^{\text{T}}-F_{2\;{\text{S}}^{2}}^{\text{T}}.\;$$ (47)

$$K_{{\text{S}}^{2}}^{\text{T}}J_{t}^{\alpha }+F_{1\;{\text{S}}^{2}}^{\text{T}}=WJ_{x}^{\beta }(II+A^{\left(a_{0}a_{3},1,\zeta_{1}\right)}+A^{\left(a_{0}a_{3},1,1\right)}-A^{\left(a_{0}a_{4},1,\zeta_{2}\right)}-A^{\left(a_{2},1,\zeta_{1}\right)}\;-C^{\left(a_{2},\zeta_{1}\right)}-C^{\left(a_{3},1\right)}+C^{\left(a_{4},\zeta_{2}\right)})+F_{3\;{\text{S}}^{2}}^{\text{T}},\;L_{{\text{S}}^{2}}^{\text{T}}J_{t}^{\alpha }+F_{2\;{\text{S}}^{2}}^{\text{T}}=VJ_{x}^{\beta }(II+A^{\left(a_{0}a_{3},1,\zeta_{1}\right)}+A^{\left(a_{0}a_{3},1,1\right)}-A^{\left(a_{0}a_{4},1,\zeta_{2}\right)}-A^{\left(a_{2},1,\zeta_{1}\right)}\;-C^{\left(a_{2},\zeta_{1}\right)}-C^{\left(a_{3},1\right)}+C^{\left(a_{4},\zeta_{2}\right)})+F_{4\;{\text{S}}^{2}}^{\text{T}},\;$$ (48)

Using the values of *W* and *V*, and suppose

$$H_{{\text{S}}^{2}\times {\text{S}}^{2}}=J_{x}^{\beta }(II-C^{\left(a_{2},\zeta_{1}\right)}-C^{\left(a_{3},1\right)}+C^{\left(a_{4},\zeta_{2}\right)}+A^{\left(a_{0}a_{3},1,\zeta_{1}\right)}\;+A^{\left(a_{0}a_{3},1,1\right)}-A^{\left(a_{0}a_{4},1,\zeta_{2}\right)}-{xA}^{\left(a_{2},1,\zeta_{1}\right)}),\;$$ (49)

Implies that

$$K_{{\text{S}}^{2}}^{\text{T}}J_{t}^{\alpha }+F_{1\;{\text{S}}^{2}}^{\text{T}}=\frac{1}{\lambda_{1}}[K_{{\text{S}}^{2}}^{\text{T}}(II-J_{t}^{\alpha }G^{\Theta_{1}})-L_{{\text{S}}^{2}}^{\text{T}}(\lambda_{2}J_{t}^{\alpha }D_{x}^{\beta }+J_{t}^{\alpha }G^{\theta_{2}})\;[4pt]-F_{1\;{\text{S}}^{2}}^{\text{T}}G^{\Theta_{1}}-F_{2\;{\text{S}}^{2}}^{\text{T}}(\lambda_{2}D_{x}^{\beta }+G^{\Theta_{2}})-S_{1}]H_{{\text{S}}^{2}\times {\text{S}}^{2}}+F_{3\;{\text{S}}^{2}}^{\text{T}},\;[4pt]L_{{\text{S}}^{2}}^{\text{T}}J_{t}^{\alpha }+F_{2\;{\text{S}}^{2}}^{\text{T}}=\frac{1}{\lambda_{4}}[L_{{\text{S}}^{2}}^{\text{T}}(II-J_{t}^{\alpha }G^{\Theta_{4}})-K_{{\text{S}}^{2}}^{\text{T}}(\lambda_{3}J_{t}^{\alpha }D_{x}^{\beta }+J_{t}^{\alpha }G^{\Theta_{3}})\;[4pt]-F_{1\;{\text{S}}^{2}}^{\text{T}}(\lambda_{3}D_{x}^{\beta }+G^{\Theta_{3}})-F_{2\;{\text{S}}^{2}}^{\text{T}}G^{\Theta_{4}}-S_{2}]H_{{\text{S}}^{2}\times {\text{S}}^{2}}+F_{4\;{\text{S}}^{2}}^{\text{T}},$$ (50)

further simplification


$$B_{1}=\frac{1}{\lambda_{1}}(II-J_{t}^{\alpha }G^{\Theta_{1}}),\;\;B_{2}=\frac{1}{\lambda_{1}}(-\lambda_{2}J_{t}^{\alpha }D_{x}^{\beta }-J_{t}^{\alpha }G^{\theta_{2}}),\;B_{3}=\frac{1}{\lambda_{1}}(-G^{\Theta_{1}}),\;\;B_{4}=\frac{1}{\lambda_{1}}(-\lambda_{2}D_{x}^{\beta }-G^{\Theta_{2}}),\;Z_{1}=\frac{1}{\lambda_{4}}(II-J_{t}^{\alpha }G^{\Theta_{4}}),Z_{2}=\frac{1}{\lambda_{4}}(-\lambda_{3}J_{t}^{\alpha }D_{x}^{\beta }-J_{t}^{\alpha }G^{\Theta_{3}}),\;Z_{3}=\frac{1}{\lambda_{4}}(-\lambda_{3}D_{x}^{\beta }-G^{\Theta_{3}}),Z_{4}=\frac{1}{\lambda_{4}}(-G^{\Theta_{4}}).\;E_{1}=\frac{1}{\lambda_{1}}S_{1},E_{2}=\frac{1}{\lambda_{4}}S_{2}.\;$$

$$k_{{\text{S}}^{2}}^{\text{T}}J_{t}^{\alpha }+F_{1}=k_{{\text{S}}^{2}}^{\text{T}}B_{1}H_{{\text{S}}^{2}\times {\text{S}}^{2}}+L_{{\text{S}}^{2}}^{\text{T}}B_{2}H_{{\text{S}}^{2}\times {\text{S}}^{2}}+F_{1}B_{3}H_{{\text{S}}^{2}\times {\text{S}}^{2}}\;+F_{2}B_{4}H_{{\text{S}}^{2}\times {\text{S}}^{2}}-E_{1}H_{{\text{S}}^{2}\times {\text{S}}^{2}}+F_{3},\;[4pt]L_{{\text{S}}^{2}}^{\text{T}}J_{t}^{\alpha }+F_{2}=L_{{\text{S}}^{2}}^{\text{T}}Z_{1}H_{{\text{S}}^{2}\times {\text{S}}^{2}}+K_{{\text{S}}^{2}}^{\text{T}}Z_{2}H_{{\text{S}}^{2}\times {\text{S}}^{2}}+F_{1}Z_{3}H_{{\text{S}}^{2}\times {\text{S}}^{2}}\;+F_{2}Z_{4}H_{{\text{S}}^{2}\times {\text{S}}^{2}}-E_{2}H_{{\text{S}}^{2}\times {\text{S}}^{2}}+F_{4}.$$ (51)

Writing the system in matrix form we get


$$\left[\begin{array}{c} k_{{\text{S}}^{2}}^{\text{T}}J_{t}^{\alpha }+F_{1}\\ L_{{\text{S}}^{2}}^{\text{T}}J_{t}^{\alpha }+F_{2} \end{array}\right]=\left[\begin{array}{c} k_{{\text{S}}^{2}}^{\text{T}}B_{1}H_{{\text{S}}^{2}\times {\text{S}}^{2}}\\ L_{{\text{S}}^{2}}^{\text{T}}Z_{1}H_{{\text{S}}^{2}\times {\text{S}}^{2}} \end{array}\right]+\left[\begin{array}{c} L_{{\text{S}}^{2}}^{\text{T}}B_{2}H_{{\text{S}}^{2}\times {\text{S}}^{2}}\\ {}[4pt]k_{{\text{S}}^{2}}^{\text{T}}Z_{2}H_{{\text{S}}^{2}\times {\text{S}}^{2}} \end{array}\right]+\left[\begin{array}{c} F_{1}k_{{\text{S}}^{2}}^{\text{T}}B_{3}H_{{\text{S}}^{2}\times {\text{S}}^{2}}\\ F_{1}k_{{\text{S}}^{2}}^{\text{T}}Z_{3}H_{{\text{S}}^{2}\times {\text{S}}^{2}} \end{array}\right]\;+\left[\begin{array}{c} F_{2}k_{{\text{S}}^{2}}^{\text{T}}B_{4}H_{{\text{S}}^{2}\times {\text{S}}^{2}}\\ F_{2}k_{{\text{S}}^{2}}^{\text{T}}Z_{4}H_{{\text{S}}^{2}\times {\text{S}}^{2}} \end{array}\right]-\left[\begin{array}{c} k_{{\text{S}}^{2}}^{\text{T}}E_{1}H_{{\text{S}}^{2}\times {\text{S}}^{2}}\\ k_{{\text{S}}^{2}}^{\text{T}}E_{2}H_{{\text{S}}^{2}\times {\text{S}}^{2}} \end{array}\right]+\left[\begin{array}{c} F_{3}\\ F_{4} \end{array}\right].$$

$$\left[\begin{array}{cc} K_{{\text{S}}^{2}}^{\text{T}} & L_{{\text{S}}^{2}}^{\text{T}} \end{array}\right]\left[\begin{array}{cc} J_{t}^{\alpha } & O\\ O & J_{t}^{\alpha } \end{array}\right]=\left[\begin{array}{cc} K_{{\text{S}}^{2}}^{\text{T}} & L_{{\text{S}}^{2}}^{\text{T}} \end{array}\right]\left[\begin{array}{cc} B_{1}H_{{\text{S}}^{2}\times {\text{S}}^{2}} & Z_{2}H_{{\text{S}}^{2}\times {\text{S}}^{2}}\\ B_{2}H_{{\text{S}}^{2}\times {\text{S}}^{2}} & Z_{1}H_{{\text{S}}^{2}\times {\text{S}}^{2}} \end{array}\right]\;+\left[\begin{array}{cc} F_{1} & F_{2} \end{array}\right]\left[\begin{array}{c} \begin{array}{cc} B_{3}H_{{\text{S}}^{2}\times {\text{S}}^{2}} & Z_{3}H_{{\text{S}}^{2}\times {\text{S}}^{2}}\\ B_{4}H_{{\text{S}}^{2}\times {\text{S}}^{2}} & Z_{4}H_{{\text{S}}^{2}\times {\text{S}}^{2}} \end{array} \end{array}\right]\;-\left[\begin{array}{cc} E_{1} & E_{2} \end{array}\right]\left[\begin{array}{cc} H_{{\text{S}}^{2}\times {\text{S}}^{2}} & O\\ O & H_{{\text{S}}^{2}\times {\text{S}}^{2}} \end{array}\right]+\left[\begin{array}{cc} F_{3} & F_{4} \end{array}\right]-\left[\begin{array}{cc} F_{1} & F_{2} \end{array}\right].$$

The above estimate is again a system of algebraic equations. It can easily be solved for the unknown coefficients vector, and hence the construction of solution is straight forward. We applied the proposed method on a variety of test problems and simulated the algorithm. The next section collects a list of examples we have solved.

## 4 Test experiments

**Test problem 1.**

$$\frac{\partial X(x,y)}{\partial y}=\frac{\partial^{2}X(x,y)}{\partial x^{2}}+\frac{1}{2}(x+y{)}^{3}X(x,y),$$ (52)

with the initial conditions


$$X(x,0)=0,\;X^{\prime }(x,0)=\frac{x^{3}}{2}-\frac{3x^{2}}{4}+\frac{x}{4},$$

We consider the following source term

$$f(x,y)=\;2\;x\;\left(y-1\right)\;\left(x-1\right)\;\left(x-\frac{1}{2}\right)+2\;x\;\left(y-\frac{1}{2}\right)\;\left(x-1\right)\;\left(x-\frac{1}{2}\right)+2\;y\;x\;\left(x-1\right)\;\left(x-\frac{1}{2}\right)\;-5\;y\;\left(y-1\right)\;\left(y-\frac{1}{2}\right)\;\left(x-1\right)\;\left(x-\frac{1}{2}\right)-5\;y\;x\;\left(t-1\right)\;\left(y-\frac{1}{2}\right)\;\left(x-1\right)\;-5\;y\;x\;\left(y-1\right)\;\left(y-\frac{1}{2}\right)\;\left(x-\frac{1}{2}\right)-\frac{y\;x\;{\left(y+x\right)}^{2}\;\left(y-1\right)\;\left(y-\frac{1}{2}\right)\;\left(x-1\right)\;\left(x-\frac{1}{2}\right)}{2}$$ (53)

The exact solution of the problem is


$$X(x,y)=y\;x\;\left(y-1\right)\;\left(y-\frac{1}{2}\right)\;\left(x-1\right)\;\left(x-\frac{1}{2}\right).$$

(see S9 Code Example 1 implementation)

**Test problem 2.**


$$\frac{\partial^{\alpha }X(x,y)}{\partial y^{\alpha }}=\frac{\partial^{\beta }X(x,y)}{\partial x^{\beta }}+2(x-y)X(x,y)+f(x,y),$$

*with the ICs and the BCs*


$$X(x,0)=x^{4},\;X^{\prime }(x,0)=x^{4},\;X(0,y)=0.7X(0.4,y)-\frac{56}{3125}e^{y},\;X(1,y)=4.5X(0.6,y)+\frac{521}{1250}e^{y}.\;$$

*with the exact solution is $X(x,y)=x^{4}e^{y}$*

*The source function is*


$$f(x,y)=x^{3}\;e^{y}(x-4)+2x^{4}\;e^{y}\;\left(y-x\right).$$

**Test problem 3.**

$$\frac{\partial^{\alpha }X(x,y)}{\partial y^{\alpha }}=3\frac{\partial^{\beta }X(x,y)}{\partial x^{\beta }}+2\frac{\partial^{\beta }Y(x,y)}{\partial x^{\beta }}+e^{\left(x+y\right)}X(x,y)+2\sin(x+y)Y(x,y)+f(x,y),\;\frac{\partial^{\alpha }Y(x,y)}{\partial y^{\alpha }}=8\frac{\partial^{\beta }X(x,y)}{\partial x^{\beta }}+3\frac{\partial^{\beta }Y(x,y)}{\partial x^{\beta }}+2\cos(x-y)X(x,y)+(x-t{)}^{2}Y(x,y)+g(x,y),\;$$ (54)

*with the ICs and the BCs*


$$X(x,0)=0,\;X(0,y)=4X(0.2,y)+(e^{\left(-2/5\right)}\sin(y)(10e^{\left(2/5\right)}-7))/5,\;X^{\prime }(x,0)=2e^{x},\;X(1,y)=3X(0.6,y)-e^{-1}\sin(y)(9e^{\left(2/5\right)}-2),\;Y(x,0)=0,\;Y(0,y)=4Y(0.2,y)+2\sin(y)-(7e^{\left(-2/5\right)}\sin(y))/5,\;Y^{\prime }(x,0)=2e^{-x},\;Y(1,y)=3Y(0.6,y)-2\;e^{-1}\;\sin\;\left(y\right)\;\left(3\;e^{\frac{2}{5}}-1\right),\;$$

*with the exact solutions*


$$X(x,y)=2\sin(y)e^{x},\;Y(x,y)=2\sin(y)e^{-x},\;$$

*the source function is*


$$f(x,y)=\frac{1}{25}2\;e^{-x}\;\sin\;\left(y\right)\;\left(45\;y-60\;x+25\;y\;x+2\right),\;g(x,y)=-2\;e^{-x}\;\sin\;\left(y\right)\;\left(8\;e^{2\;x}-2\;y\;x+2\;\cos\;\left(y-x\right)\;e^{2\;x}+y^{2}+x^{2}-2\right).\;$$

## 5 Results and discussion

After applying the algorithm to the test problem. We observed that the approximate solution matches very well with the exact solution of the problem. We approximate the solution of the Example 1, using different choices of the scale level. We observed that at a very small scale *M* = 4, the exact and approximate solution matches very well. In order to measure the accuracy, we used two types of measures, absolute error and relative error. They are defined by the following formula.


$$E^{m}(X)=|X(x,y)-X_{m}(x,y)|$$

$$E_{re}^{m}(X)=\frac{\left|X(x,y)-X_{m}(x,y)\right|}{max(X(x,y))}$$

We also measured the order of convergence of the relative error of the algorithm. We used the following measure for the order of convergence.

$$\text{Rate}=\frac{\log\left(\frac{E_{re}^{m}(X)}{E_{re}^{m+1}(X)}\right)}{\log\left(\frac{M}{M+1}\right)}$$ (55)

**Fig 1:**
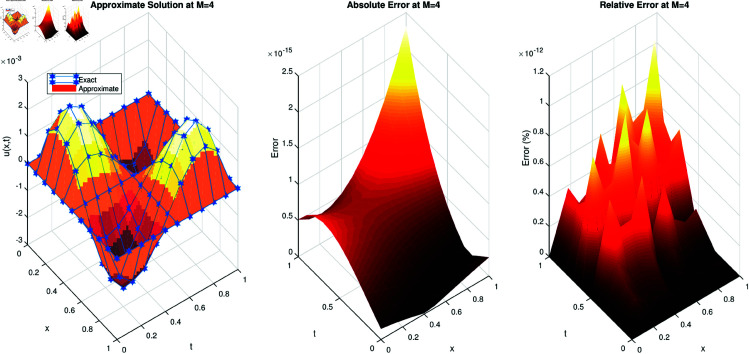
(a) Comparison of the approximate solution with the exact solution for the example 1. (b) Absolute error of example 1. (c) Relative error of example 1.

In Fig 1, we present three graphs. The first graph illustrates a comparison between the exact and approximate solutions of the problem. The second graph displays the absolute difference between these solutions, where it is observed that the absolute difference remains consistently below $1.2\times 10^{-14}$. The third graph represents the relative error, which is notably smaller than $1.2\times 10^{-13}$. These accuracy levels highlight the remarkable efficiency of the proposed method.

**Fig 2:**
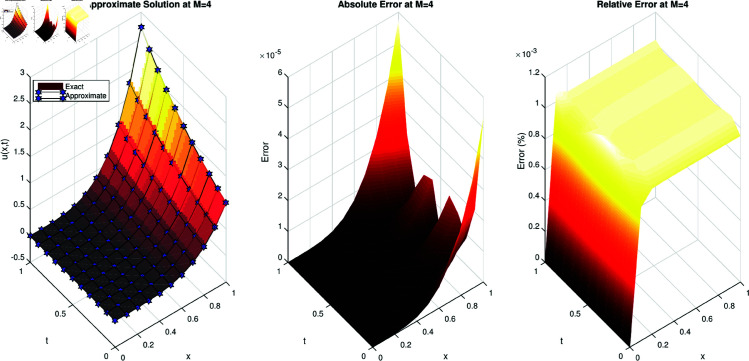
(a) Comparison of the approximate solution with the exact solution for the example 2. (b) Absolute error of example 2. (c) Relative error of example 2.

A similar comparison for Example 2 is depicted in Fig 2. Both the absolute error and relative error demonstrate a high degree of accuracy.

For Test Problem 3, we initially fixed the order of the derivative to check that whether the scheme works for the integer orders or not and then ran the algorithm across different scale levels. Verifying the convergence of the proposed method often requires analyzing the behavior of the solution at varying scale levels. In this study, we utilized scale levels ranging from 2 to 7 and constructed approximate solutions *X*(*x*,*y*) and *Y*(*x*,*y*), subsequently measuring both absolute and relative errors.

**Fig 3:**
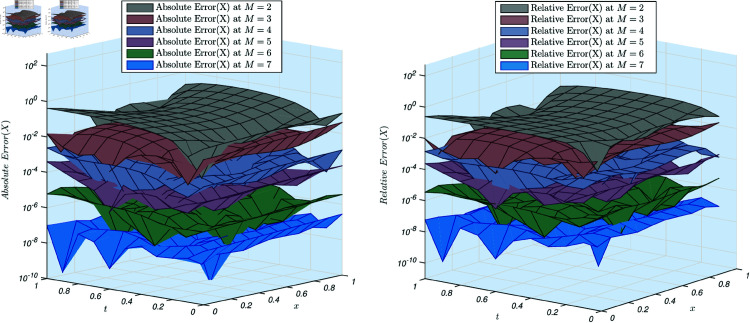
(a) Absolute error of the example 3, X(x,y) for scale level ranging from 2 to 7. (a) Relative error of the example 3, for scale level ranging from 2 to 7.

Fig 3 illustrates the absolute and relative errors for the solution *X*(*x*,*y*). It is observed that both errors decrease as the scale level increases. Notably, at scale level 4, both errors fall below 10^−6^. Additionally, the maximum value of the solution across the interval ensures minimal variation between the absolute and relative errors. This decreasing trend in both errors confirms the sufficient convergence and robustness of the proposed method.

**Fig 4:**
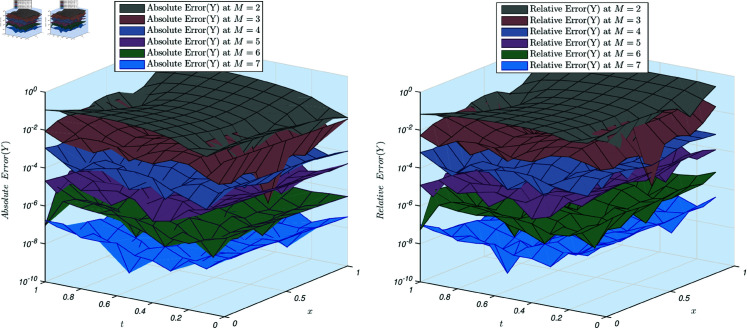
(a) Absolute error of the example 3, Y(x,y) for scale level ranging from 2 to 7. (a) Relative error of the example 3, for scale level ranging from 2 to 7.

Fig 4 shows the absolute and relative errors for the solution *Y*(*x*,*y*). The same results are observed for this solution.

**Fig 5:**
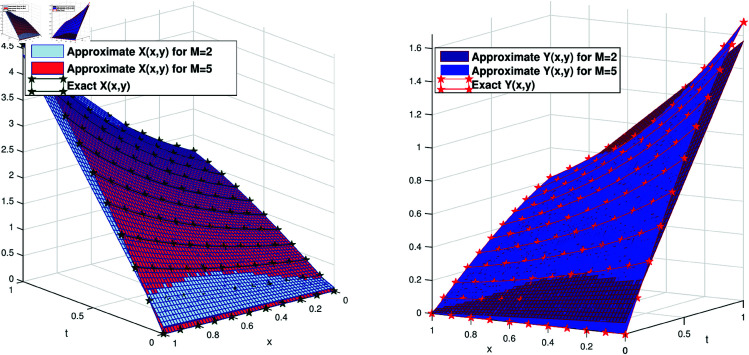
(a) Comparison of the approximate solution X(x,y) with the exact solution of the problem 3. (a) Comparison of the approximate solution *Y*(*x*,*y*) with the exact solution of the problem 3.

For the third problem, we also displayed the comparison of the exact and approximate solution of the exact solution with the approximate solution at different scale levels. We plotted the exact solution of the problem and approximate solution for *M* = 2 and *M* = 5. We see that at *M* = 2 the approximate solution is a little far from the exact solution while for *M* = 5 both the solution matches very well with each other. This phenomena is illustrated in Fig 5(a) and 5(b).

**Fig 6:**
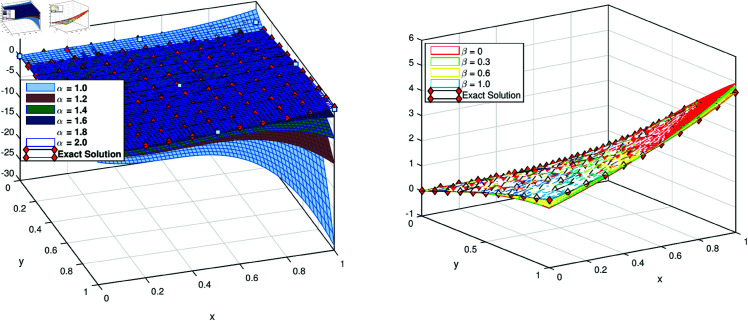
Approximate X(x,y) for different value of α and β for test problem 3.

**Fig 7:**
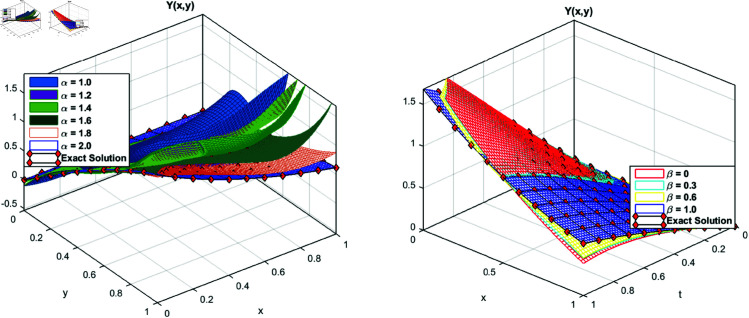
Approximate Y(x,y) for different value of α and β for test problem 3.

To check that how the proposed method works for the fractional orders, we plot the results for the different values of orders α and β. The exact solution for the fractional values are not know explicitly, however it is one of the well know property of fraction order equation, that as the order α as approach to 1, the solution for fraction values approaches to the exact solution for the integer value. We verified this property of the scheme, we first fixed the β=1 and applied a variation to the order α. We observe that the solution *X*(*x*,*y*) approaches to the exact solution at integer order as α→1. This phenomena is illustrated in Fig 6(a). We performed variational approach for a variation of β. The results for different values of β is displayed in (b) part of the same figure. The parametric results of the solution *Y*(*x*,*y*) is shown in the Fig 7. We observe that as the order of the derivative approaches the integer value the solution of the problem approaches the solution of the integer order problem.

To check the convergence of scheme quantitatively, we present the results in numerical form in Tables 1 and 2. The absolute error and relative error are presented in Table 1. The rate of convergence of the relative error of the algorithm is displayed in Table 2. We observe that the relative error convergence rates are consistently between 1.0 and 1.5, which means the error is reducing by roughly an order of magnitude of the increments in scale level *M*.

**Table 1:** Comparison of absolute and relative errors.

M	$\left|X_{e}\right|$	$\left|X_{r}\right|$	$\left|Y_{e}\right|$	$\left|Y_{r}\right|$
2	0.143336345356156	0.091036996045764	0.0475454388489818	0.0790134798807663
3	0.0137757903818371	0.00686218002396599	0.00522486628882513	0.00819017052153703
4	0.000869609761224223	0.00041165731364211	0.000267823315185095	0.000406722855545444
5	8.01822807194304e-05	3.97650234029796e-05	1.94096492998091e-05	3.47077160661949e-05
6	2.23555797723605e-06	1.26986487224306e-06	1.03156338272638e-06	1.57619941201018e-06
7	8.85849689633335e-08	4.80265435553792e-08	3.33554500726063e-08	5.58566549617468e-08

**Table 2:** Rate of convergence for relative errors.

Between M	Rate for |*X*_*r*_|	Rate for |*Y*_*r*_|
2 and 3	1.122	0.985
3 and 4	1.223	1.304
4 and 5	1.016	1.070
5 and 6	1.497	1.342
6 and 7	1.423	1.451

## 6 Conclusion and future work

In this paper, we presented a highly efficient numerical method for solving fractional-order partial differential equations and their coupled systems. Our method is based on shifted Legendre polynomials. The newly developed operational matrices effectively transformed the complex fractional-order systems into a set of algebraic Sylvester-type equations. The proposed scheme has the ability to handle two-point nonlocal boundary conditions. By solving a series of numerical experiments, we demonstrated that the proposed method achieves acceptable accuracy, with both absolute and relative errors decreasing with the increase in scale level. For future research, several potential directions can be explored. One possibility is to extend the current method to handle more complex boundary conditions, including time-dependent or mixed-type constraints. Applying the scheme to higher-dimensional fractional differential equations is also a good area of research. Additionally, hybrid computational techniques that combine spectral methods with other numerical discretization approaches could be investigated to enhance flexibility and efficiency.

## Supporting information

S1 Code MATLAB code for Legendre polynomial calculation. (PDF)

S2 Code MATLAB code for two dimensional Legendre polynomial. (PDF)

S3 Code MATLAB code for computing coefficients using Legendre polynomials. (PDF)

S4 Code MATLAB code for computing fractional-order derivative for single dimension. (PDF)

S5 Code MATLAB code for constructing a fractional-order derivative matrix. (PDF)

S6 Code MATLAB code for fractional-order integral matrix for one dimension. (PDF)

S7 Code MATLAB code for constructing a fractional-order integral matrix in two dimensions. (PDF)

S8 Code MATLAB code for variable coefficient fractional-order derivative. (PDF)

S9 Code MATLAB code for fractional PDE approximation and error analysis for example 1. (PDF)
