# Correction: A novel method for approximate solution of two point non local fractional order coupled boundary value problems

**DOI:** 10.1371/journal.pone.0335237

**Published:** 2025-10-22

**Authors:** Lahoucine Tadoummant, Hammad Khalil, Rachid Echarghaoui, Sarah Aljohani, Nabil Mlaiki

The third author’s name is spelled incorrectly. The correct name is: Rachid Echarghaoui. The correct citation is: Tadoummant L, Khalil H, Echarghaoui R, Aljohani S, Mlaiki N (2025) A novel method for approximate solution of two point non local fractional order coupled boundary value problems. PLoS One 20(7): e0326101. https://doi.org/10.1371/journal.pone.0326101.
