# Supplementary material for: A novel method for approximate solution of two point non local fractional order coupled boundary value problems
Source: PLoS One. 2025 Jul 2;20(7):e0326101. doi: 10.1371/journal.pone.0326101 (PMC12221080; doi:10.1371/journal.pone.0326101)
Supplement: S1 Code — (PDF) [file pone.0326101.s001.pdf]

Supporting Information: MATLAB Code for Fractional-Order PDE Solutions

S1 Code: MATLAB code for Legendre polynomial calculation

```
function P=leg_poly(m,x)
syms k ;
for i=0:m-1;
    p1=((-1)^(i+k))*factorial(i+k);
    p2=factorial(i-k)*(factorial(k))^2;
    p3=(p1/p2)*(x^k);
    p4=symsum(p3,k,0,i);
    P(i+1,1)=p4;
end
```

Listing 1: leg_poly.m
