# Supplementary material for: A novel method for approximate solution of two point non local fractional order coupled boundary value problems
Source: PLoS One. 2025 Jul 2;20(7):e0326101. doi: 10.1371/journal.pone.0326101 (PMC12221080; doi:10.1371/journal.pone.0326101)
Supplement: S2 Code — (PDF) [file pone.0326101.s002.pdf]

Supporting Information: MATLAB Code for Fractional-Order PDE Solutions

S2 Code: MATLAB code for two dimensional Legendre polynomial

```
function PPP=l_poly(m,x,t)
P=leg_poly(m,x);
P1=leg_poly(m,t);
for r=1:m^2;
[i,j]=index_file(m,r);
PP(r)=P(i+1)*P1(j+1);
PPP(r,1)=PP(r);
end
end
```

Listing 1: l_poly.m
