# Supplementary material for: A novel method for approximate solution of two point non local fractional order coupled boundary value problems
Source: PLoS One. 2025 Jul 2;20(7):e0326101. doi: 10.1371/journal.pone.0326101 (PMC12221080; doi:10.1371/journal.pone.0326101)
Supplement: S3 Code — (PDF) [file pone.0326101.s003.pdf]

Supporting Information: MATLAB Code for Fractional-Order PDE Solutions

S3 Code: MATLAB code for computing coefficients using Legendre polynomials

```
function C=l_coe(f,m,x,t)
P=l_poly(m,x,t);
Pf=f*P;
PFI=int(int(Pf,x,0,1),t,0,1);
for r=1:m^2;
[i,j]=index_file(m,r);
PFIC(r)=double(PFI(r)*(2*(i+1)-1)*(2*(j+1)-1));
end
C=(PFIC);
```

Listing 1: l_coe.m
