# Supplementary material for: A novel method for approximate solution of two point non local fractional order coupled boundary value problems
Source: PLoS One. 2025 Jul 2;20(7):e0326101. doi: 10.1371/journal.pone.0326101 (PMC12221080; doi:10.1371/journal.pone.0326101)
Supplement: S4 Code — (PDF) [file pone.0326101.s004.pdf]

Supporting Information: MATLAB Code for Fractional-Order PDE Solutions

S4 Code: MATLAB code for computing fractional-order derivative for single dimension

```
function P=Lsin_der(alpha,m)
syms k l
a=0;
b=0;
eta=1;
d=floor(alpha);
for i=0:m-1;
for j=0:m-1;
aa=((-1)^(i-k)) * gamma(i+b+1) * gamma(i+k+a+b+1);
bb= gamma(k+b+1) * gamma(i+a+b+1) * gamma(i-k+1)* gamma(k-alpha+1)
    ;
cc=((-1)^(j-l)) * gamma(j+l+a+b+1) * gamma(a+1) * gamma(l+k-alpha+
    b+1) ...
*(2*j+a+b+1)*gamma(j+1) *(eta^(-alpha));
dd=gamma(j+a+1) *gamma(l+b+1) *gamma(j-l+1) *gamma(l+1) ...
*gamma(l+k+a+b-alpha+2);
P(i+1,j+1)=double(symsum(((aa/bb) * symsum((cc/dd),l,0,j)),k,d,i))
    ;
end
end
```

Listing 1: Lsin_der.m
