# Supplementary material for: A novel method for approximate solution of two point non local fractional order coupled boundary value problems
Source: PLoS One. 2025 Jul 2;20(7):e0326101. doi: 10.1371/journal.pone.0326101 (PMC12221080; doi:10.1371/journal.pone.0326101)
Supplement: S5 Code — (PDF) [file pone.0326101.s005.pdf]

Supporting Information: MATLAB Code for Fractional-Order PDE Solutions

S5 Code: MATLAB code for constructing a fractional-order derivative matrix

```
function Q=L2_xder_mat(alpha,m);
J=m-1;
syms k i j l;
size=(m^2);
A=creat_index(m);
Ab=Lsin_der(alpha,m);

for nn=1:size;
a=A(nn,1);
b=A(nn,2);

for mm=1:size;
d=A(mm,1);
e=A(mm,2);
if b==e;
Qa(nn,mm)=Ab(a,d);
else
Qa(nn,mm)=0;
end
end
end

Q=double(Qa);
```

Listing 1: L2_xder_mat.m
