# Supplementary material for: A novel method for approximate solution of two point non local fractional order coupled boundary value problems
Source: PLoS One. 2025 Jul 2;20(7):e0326101. doi: 10.1371/journal.pone.0326101 (PMC12221080; doi:10.1371/journal.pone.0326101)
Supplement: S8 Code — (PDF) [file pone.0326101.s008.pdf]

Supporting Information: MATLAB Code for Fractional-Order PDE Solutions

S8 Code: MATLAB code for variable coefficient fractional-order derivative

```
function Q=var_xderivtive(pie,alpha,m)
% pie is the variable coefficients of the differential components
% alpha is the order of derivative with respect to x

syms x t

if alpha==0;
P=eye(m^2);
else
P=L2_xder_mat(alpha,m);
end

B=l_poly(m,x,t);
D=pie*B;

for i=1:m^2;
Qa(i,:)=l_coe2(D(i),m);
end

Q=double(P*Qa);
```

Listing 1: var_xderivtive.m
