# Supplementary material for: A novel method for approximate solution of two point non local fractional order coupled boundary value problems
Source: PLoS One. 2025 Jul 2;20(7):e0326101. doi: 10.1371/journal.pone.0326101 (PMC12221080; doi:10.1371/journal.pone.0326101)
Supplement: S9 Code — (PDF) [file pone.0326101.s009.pdf]

Supporting Information: MATLAB Code for Fractional-Order PDE Solutions

S9 Code: MATLAB code for fractional PDE approximation and error analysis for example 1

```
clear all;
clc;

% Define symbolic variables
syms x t;

% Parameters
alpha = 2;
beta = 1;
lambda = 5;
phi2 = (1/2)*(x + t)^3;
U = (t-1)*(x-1)*(x-0.5)*(t-0.5)*x*t;

% Initial and boundary conditions
f1 = subs(U, t, 0);
f2 = subs(diff(U, t), t, 0);
f3 = f1 + f2 * t;

% PDE
f = diff(U, t, 2) - lambda * diff(U, x, beta) - phi2 * U;

% Approximation parameters
for m = 3:5;
OME = l_poly(m, x, t);
F1 = l_coe(f3, m, x, t);
F2 = l_coe(f, m, x, t);
Db = L2_xder_mat(beta, m);
P = L2_tint_mat(alpha, m);
Q2 = var_xderivtive(phi2, 0, m);

% System Matrices
A = 1;
B = -double(lambda * P * Db + P * Q2);
C = -double(lambda * F1 * Db + F1 * Q2 + F2);

```

```
% Solve Lyapunov equation
X = lyap(A, B, C);

% Approximate Solution
Uapp = X * P * OME + F1 * OME;

dom = 0:0.1:1;
UE = double(subs(subs(U, x, dom), t, transpose(dom)));
UApp_Eval = double(subs(subs(Uapp, x, dom), t, transpose(dom)));

% Absolute Error
AbsError = abs(UE - UApp_Eval);

% Relative Error (Avoid division by zero)
RelError = abs((UE - UApp_Eval) ./ UE + 0.001);
RelError(isnan(RelError) | isinf(RelError)) = 0;

% Visualization
figure('Name', 'Solution and Error Analysis', 'NumberTitle', 'off', 'Color', 'w');

subplot(1, 3, 1);
mesh(dom, dom, UE, 'FaceAlpha', 0.7);
hold on;
surf(dom, dom, UApp_Eval, 'FaceAlpha', 0.7);
title('Exact vs Approximate Solution at M=4');
xlabel('x'); ylabel('t'); zlabel('u(x,t)');
legend('Exact', 'Approximate');
colormap('parula'); shading interp;

subplot(1, 3, 2);
surf(dom, dom, AbsError);
title('Absolute Error at M=4');
xlabel('x'); ylabel('t'); zlabel('Error');
colormap('jet'); shading interp;
colorbar;

subplot(1, 3, 3);
surf(dom, dom, RelError);
title('Relative Error at M=4');
xlabel('x'); ylabel('t'); zlabel('Error (%)');
colormap('hot'); shading interp;
colorbar;
end
```

Listing 1: example1_fractional_pde.m
